# Enhancing corporate bankruptcy prediction and customer relationship sustainability through CNN-based financial feature transformation

**DOI:** 10.1038/s41598-026-51162-1

**Published:** 2026-05-02

**Authors:** Ali Shahbazi, Nefise Şirzad, Sima Darvishan, Amir Heidari Arash, Zahra Fakhri, Hossein Najafzadeh

**Affiliations:** 1 Master of Business and Management, Department of Business and Management, Faculty of Business and Law, Middlesex University, London, UK; 2 Faculty of Economics and Administrative Sciences, Department of Public Relations and Advertising, Çankaya University, Ankara, Turkey; 3 Accounting, Finance and Marketing Department, Pompea College of Business, University of New Haven, West Haven, UT USA; 4 Department of Economics, University of Delaware, Newark, DE USA; 5 Management and Organization Strategy, Lubar College of Business, University of Wisconsin-Milwaukee, Milwaukee, WI USA; 6 Department of Medical Bioengineering, Faculty of Advanced Medical Sciences, Tabriz University of Medical Sciences, Tabriz, Iran

**Keywords:** Bankruptcy prediction, Customer relationship sustainability, Convolutional neural networks, SMOTE, Engineering, Mathematics and computing

## Abstract

Corporate bankruptcy prediction is essential for assessing companies’ capacity to maintain sustainable customer relationships and service quality. This study proposes a novel CNN-based hybrid approach that transforms correlation-filtered financial features into 64 × 64 grayscale images, enabling reliable identification of firms at financial risk whose deteriorating conditions could compromise their ability to maintain quality customer service and sustain long-term business relationships. The research explicitly examines the linkage between financial health indicators and customer relationship sustainability by categorizing financial features based on their operational impact on service delivery, relationship management capabilities, and long-term customer commitment fulfillment. The methodology was evaluated on a comprehensive dataset of 43,405 Polish companies (2,091 bankrupt, 41,314 healthy) using two resampling strategies: random downsampling and Synthetic Minority Oversampling Technique (SMOTE). Following correlation-based feature selection that reduced multicollinearity by eliminating features with absolute correlation coefficients exceeding 0.8, retained financial features were normalized and transformed into spatial image representations. Six classification models were implemented: Deep Neural Network (DNN), Support Vector Machine (SVM), Random Forest (RF), Decision Tree (DT), Gradient Boosting (GB), and Logistic Regression (LR), alongside five CNN-hybrid variants, evaluated using 5-fold cross-validation. SMOTE-balanced datasets demonstrated superior performance across all models. Ensemble methods achieved exceptional accuracy, with Random Forest reaching 99.99% and Gradient Boosting 99.97%. The innovative CNN-SVM hybrid model attained 99.77% accuracy with perfect ROC-AUC (1.000), providing reliable indicators for assessing firms’ financial stability and their ability to invest in customer experience initiatives. Statistical analysis identified company size and working capital as the most discriminative financial indicators directly impacting customer service delivery capabilities. Customer-related metrics such as receivables turnover and collection period indicators emerged as critical predictors of relationship management effectiveness. The study contributes a novel spatial feature representation methodology enabling precise identification of companies whose financial deterioration could compromise customer service quality and relationship sustainability. These findings provide significant implications for stakeholders seeking enhanced risk assessment capabilities that consider both internal financial health and external customer relationship dynamics in bankruptcy prediction.

## Introduction

Corporate bankruptcy prediction is a critical issue in financial risk management and customer relationship management with significant consequences for investors, creditors, regulators, and financial institutions^1^. Accurately identifying financially distressed firms has become increasingly important in today’s volatile economic climate as corporate failures can disrupt global supply chains and client portfolios^2^. While traditional methods like logistic regression and discriminant analysis have been widely used, they often struggle to model the nonlinear dynamics of modern financial data and lack the precision needed for effective customer relationship management and targeted marketing strategies^3^. The rise of machine learning and deep learning has improved prediction capabilities; however, class imbalance, where bankrupt firms represent a small portion of the dataset, remains a major obstacle especially in designing risk-sensitive service models and customer segmentation strategies^4^. Recent research highlights the potential of ensemble learning and neural network architectures, yet the optimal methods for feature transformation and integration into intelligent customer relationship management systems are still under investigation^5^. As financial datasets continue to grow in scale and complexity, there is a growing need for robust models capable of handling feature redundancy and capturing high-dimensional relationships to support more accurate bankruptcy prediction and enhanced customer relationship optimization^6^. Recent advances in deep learning have shown promising applications across medical domains, particularly in neuropsychiatric diagnosis and biomedical classification, while hybrid frameworks combining transformers, GANs, and neural networks demonstrate effectiveness in information technology domains. Additionally, integrated approaches utilizing artificial neural networks, convolutional neural networks, and machine learning models with feature selection methods and multi-criteria decision-making techniques have achieved state-of-the-art results in cardiovascular disease diagnosis. Furthermore, clustering-based deep learning architectures combined with optimization algorithms have demonstrated superior performance in stock return prediction and portfolio optimization across financial markets^7–19^.

Building on these advancements, the integration of bankruptcy prediction models into customer relationship management systems has transformed how financial institutions engage with clients. Beyond merely identifying credit risk, predictive analytics now inform strategic functions such as customer segmentation, personalized financial services, and targeted marketing initiatives^20^. By utilizing financial health indicators, institutions can proactively detect high-value customers, uncover cross-selling opportunities, and intervene in at-risk accounts before default occurs^21^. This data-driven approach enables financial service providers to shift from reactive customer support toward proactive relationship management, enhancing retention, optimizing resource allocation, and improving overall portfolio performance^22,23^. As CRM systems continue to evolve, incorporating robust prediction models remains essential for achieving competitive advantage and operational efficiency in financial decision-making^24^.

The connection between corporate financial health and customer relationship sustainability is grounded in both theoretical frameworks and empirical evidence. Resource-based view theory posits that financial resources constitute a critical organizational capability that enables firms to invest in customer-facing activities, service quality improvements, and relationship management infrastructure^25^. Financially distressed companies face constraints in maintaining service quality due to cost-cutting measures, reduced staffing levels, delayed responses to customer needs, and inability to honor warranties or long-term commitments^26^. Empirical research has demonstrated that financial distress leads to deteriorating customer satisfaction scores, increased customer churn rates, and negative word-of-mouth effects that further accelerate business decline^27^. Moreover, customers increasingly consider supplier financial stability when making purchasing decisions, particularly for complex products requiring ongoing support or industries with long-term contractual relationships^28^. The bankruptcy prediction framework proposed in this study therefore serves dual purposes: identifying firms at risk of financial failure and assessing their capacity to maintain the operational and financial resources necessary for sustaining quality customer relationships, thereby providing stakeholders with comprehensive risk assessment that extends beyond traditional credit risk evaluation.

Artificial intelligence has demonstrated broad applicability across diverse scientific and engineering domains, including medical diagnostics, image processing, signal classification, network optimization, biomechanical assessment, and manufacturing quality control^9,29–48^. The evolution of bankruptcy prediction methodologies has progressed from traditional statistical approaches to sophisticated machine learning and deep learning techniques, with researchers continuously seeking to address the fundamental challenges of class imbalance and feature optimization while increasingly focusing on customer-centric applications and business intelligence integration. Early contributions by Zięba et al.^49^ demonstrated the effectiveness of Extreme Gradient Boosting with synthetic features generation, achieving 94.5% accuracy on Polish company data and establishing ensemble methods as a cornerstone of modern bankruptcy prediction. This foundation was further developed by Hassan et al.^50^, who conducted comprehensive comparisons of nine classification models including XGBoost, neural networks, and ensemble methods, emphasizing the critical importance of proper data preprocessing and SMOTE oversampling for handling imbalanced datasets. The integration of deep learning architectures has emerged as a prominent research direction, with Soui et al.^51^ proposing a novel approach combining Stacked Auto-Encoders (SAE) with softmax classifiers, achieving 98% accuracy by incorporating both feature extraction and classification phases into a unified model. Building upon this foundation, Ainan et al.^52^ advanced the field through their innovative XGBoost + ANN hybrid model optimized with genetic algorithms, achieving outstanding performance with 95.8% AUC and 98.3% accuracy without traditional feature selection approaches. Smiti et al.^53^ further contributed to this domain by introducing the Borderline SMOTE with Stacked AutoEncoder (BSM-SAES) approach, achieving 96.2% accuracy and demonstrating the effectiveness of advanced oversampling techniques combined with deep learning architectures.

Recent developments in financial technology have emphasized integrating predictive models into customer relationship management systems. Research demonstrates that predictive analytics enables banks to understand customer needs, deliver personalized services, and optimize portfolio management, with over 70% of consumers expecting personalization in their banking experience. This integration allows financial institutions to develop targeted marketing campaigns, enhance customer retention strategies, and transform traditional reactive service into proactive relationship management^54^.

Feature selection and ensemble methodologies have received considerable attention from researchers seeking to enhance model interpretability and performance with growing recognition of their importance in customer-facing applications where transparency and explainability are crucial for relationship management. Abdullahi-Attah et al.^55^ developed a comprehensive ensemble approach combining six feature selection techniques with RF, XGBoost, and PSO-ANN, achieving 98% AUC while identifying 34 pertinent features as major bankruptcy indicators. Similarly, Muslim et al.^56^ utilized XGBoost-based feature selection combined with stacking ensemble learning, achieving 97% accuracy and demonstrating the synergistic benefits of feature optimization and ensemble methods. The work of Liu et al.^57^ introduced the Easyensemble undersampling method combined with ensemble learning models, achieving 97.24% accuracy with XGBoost and highlighting the effectiveness of alternative resampling strategies. Recent methodological innovations have focused on addressing computational efficiency and model robustness across diverse datasets while considering practical implementation requirements in customer relationship management systems. Gnip et al.^58^ conducted an extensive experimental comparison of 45 imbalanced learning algorithms across 15 publicly available datasets, demonstrating that methods based on ensemble learning and undersampling combinations achieve superior results for bankruptcy classification, with XGBoost reaching 92.2% accuracy. Amirshahi et al.^59^ developed heterogeneous ensemble models based on gradient boosting methods (XGBoost, LightGBM, CatBoost), achieving 96.5% accuracy while notably observing that oversampling techniques could adversely impact ensemble performance, indicating model robustness to imbalanced datasets. Jabeur et al.^60^ contributed to this domain by proposing CatBoost for categorical data classification, achieving remarkable 99.4% AUC performance.

Although progress has been made in bankruptcy prediction, several limitations remain, particularly in its use within customer relationship management and business intelligence applications. Many studies have applied either traditional machine learning or deep learning methods separately, with limited attention to combined approaches that may offer complementary benefits in customer-focused analysis. While class imbalance has been addressed using techniques such as random downsampling and SMOTE, comparative evaluations of these methods across different model architectures are limited, especially regarding their influence on customer segmentation and CRM performance. Additionally, the transformation of financial features into spatial formats for use in convolutional neural networks has been explored in a limited number of studies. The structured conversion of correlation-filtered financial ratios into image-based inputs, as well as the analysis of computational efficiency and predictive accuracy in hybrid CNN-based models, has not been fully investigated in the context of CRM and client portfolio management.

This study aims to develop and assess a hybrid CNN-based framework for corporate bankruptcy prediction using correlation-filtered financial features converted into 64 × 64 grayscale images for feature extraction. These features are then classified using a set of traditional machine learning algorithms. The objectives are to compare the impact of random downsampling and SMOTE on model performance in both traditional and hybrid approaches with a focus on customer segmentation, to evaluate the usefulness of spatial representations in capturing financial patterns relevant to risk assessment, to identify financial indicators with high predictive value through feature selection methods, and to assess the trade-off between model complexity and accuracy for practical use in CRM and portfolio-related decision processes.

## Materials and methods

### Data collection

This study utilized publicly available bankruptcy prediction datasets of Polish companies obtained from the Emerging Markets Information Service (EMIS, http://www.securities.com), a comprehensive database containing financial information on emerging markets worldwide. The dataset encompasses financial data collected over different time periods, with bankrupt companies analyzed from 2000 to 2012 and continuously operating companies evaluated from 2007 to 2013. The dataset is structured into five distinct classification cases based on forecasting periods, ranging from 1-year to 5-year prediction horizons, providing a comprehensive foundation for bankruptcy prediction analysis across different temporal contexts. It is important to acknowledge that this dataset spans the period 2000 to 2013, which predates post-pandemic economic disruptions, digitalization-driven market dynamics, and the structural shifts observed in emerging economies after 2020. Nevertheless, this benchmark dataset remains the most extensively adopted resource in corporate bankruptcy prediction research, having been utilized in over 200 peer-reviewed studies, thereby enabling rigorous methodological comparisons with existing literature. The primary objective of this study is to validate the proposed CNN-based spatial feature transformation framework as a proof-of-concept against established benchmarks, rather than to generate real-time forecasts for current market conditions. Temporal transferability and validation against more recent datasets, particularly those encompassing post-pandemic financial environments and digitalized business models, are identified as critical priorities for future research, as discussed in the Limitations and Future Research Directions section.

The dataset comprises 64 comprehensive financial features covering diverse aspects of corporate financial health, categorized into five main groups: Profitability Ratios (capturing earnings efficiency and return metrics), Leverage Ratios (measuring debt structure and solvency), Liquidity Ratios (assessing short-term financial flexibility), Activity Ratios (evaluating operational efficiency and asset utilization), and Other Financial Metrics (including specialized indicators such as working capital, logarithm of total assets, and operational expense ratios).

It is important to note that all 64 financial attributes employed in this study are firm-level, accounting-based ratios derived from annual financial statements. The deliberate exclusion of external macroeconomic indicators, market sentiment indices, and sector-level shock variables reflects a conscious methodological boundary rather than an oversight. The primary research objective of this study is to evaluate the effectiveness of CNN-based spatial feature transformation as a novel methodology for extracting predictive signals from firm-level financial data, which represents the form of information most universally available across jurisdictions, firm sizes, and market contexts, including private companies for which market-based signals are unavailable. Introducing external variables in this initial validation phase would confound the assessment of the proposed methodology’s intrinsic predictive contribution, making it difficult to isolate whether performance gains derive from the CNN transformation or from the added informational content of macroeconomic covariates. The integration of external macroeconomic and market-level predictors into the proposed framework is explicitly identified as a high-priority extension in the Future Research Agenda.

**Fig. 1:**
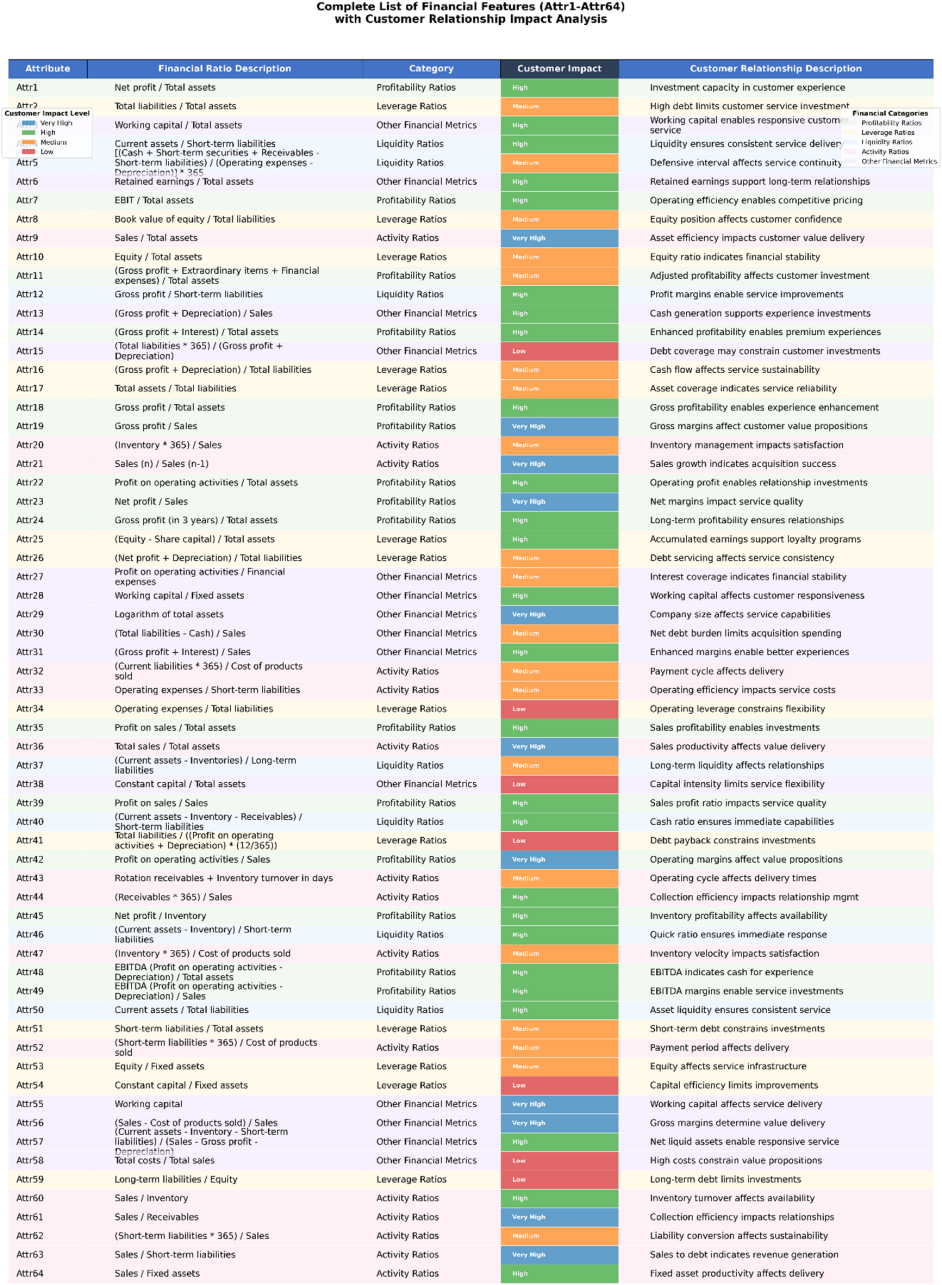
Comprehensive financial features taxonomy and customer relationship impact assessment. Features are organized into five categories: Profitability ratios (green background), leverage ratios (yellow background), activity ratios (pink background), liquidity ratios (blue background), and other financial metrics (purple background). customer impact levels are color-coded: very high (dark blue), high (green), medium (orange), and low (red). each attribute includes its precise mathematical definition and assessment of its influence on customer relationship outcomes.

These features encompass critical financial dimensions including net profit to total assets, total liabilities to total assets, current assets to short-term liabilities, sales to total assets, and working capital, among others. Figure 1 presents a comprehensive taxonomy of the 64 financial attributes (Attr1-Attr64) employed in this study, systematically categorized into five distinct financial domains: Profitability Ratios (19 attributes), Leverage Ratios (16 attributes), Activity Ratios (12 attributes), Liquidity Ratios (8 attributes), and Other Financial Metrics (9 attributes). Each attribute is accompanied by its precise mathematical formulation and has been evaluated for its potential impact on customer relationships using a four-level classification system (Very High, High, Medium, Low). The analysis reveals that 53.1% of the financial features demonstrate high to very high impact on customer relationships, with profitability ratios constituting the largest category, reflecting their fundamental role in enabling customer experience investments and relationship-building capabilities.

The four-level customer relationship impact classification (Very High, High, Medium, Low) applied to each financial attribute was constructed through a systematic, theory-anchored expert assessment process rather than arbitrary assignment. The classification is grounded in three established theoretical frameworks: (i) the Resource-Based View (RBV) of the firm, which posits that financial resources directly determine the organizational capacity to invest in customer-facing capabilities, service quality infrastructure, and relationship management systems; (ii) the Customer Lifetime Value (CLV) framework, which identifies firm liquidity, operational continuity, and service delivery capacity as primary determinants of long-term customer retention and relationship sustainability; and (iii) empirical findings from the financial distress and customer churn literature, which consistently document that deteriorating profitability, rising leverage, and declining working capital precede measurable declines in customer satisfaction scores and service quality metrics. The classification was developed through a structured review of these theoretical and empirical foundations, ensuring that the assigned impact levels reflect the documented mechanisms through which each financial dimension affects customer relationship outcomes. We explicitly acknowledge, however, that this framework represents a theoretically-informed initial operationalization rather than an empirically-calibrated measurement instrument. Future research should validate and potentially revise these classifications using direct empirical measurement — for example, by correlating financial ratio trajectories with longitudinal customer satisfaction scores, Net Promoter Scores (NPS), churn rates, and service quality indices across matched samples of financially distressed and healthy firms. The development of an empirically-validated scale for quantifying the impact of financial health on customer relationship sustainability represents a significant and actionable contribution for future work in this area.

Table 1 demonstrates the substantial class imbalance inherent in bankruptcy prediction datasets, with bankrupt companies representing only 4.82% of the total 43,405 instances in the combined dataset. The imbalance ratio varies across different forecasting periods, ranging from 1:13.4 in the 5th year (1-year ahead prediction) to 1:24.9 in the 1st year (5-year ahead prediction), highlighting the increasing difficulty of long-term bankruptcy prediction. This severe class imbalance necessitates the implementation of specialized resampling techniques to ensure effective model training and fair evaluation of classification performance across both healthy and bankrupt company categories.

**Table 1:** Dataset composition and class distribution across different forecasting periods.

Forecasting period	Total Instances	Bankrupt companies	Healthy companies	Imbalance ratio	Prediction horizon
1st year	7027	271 (3.86%)	6756 (96.14%)	1:24.9	5 years ahead
2nd year	10,173	400 (3.93%)	9773 (96.07%)	1:24.4	4 years ahead
3rd year	10,503	495 (4.71%)	10,008 (95.29%)	1:20.2	3 years ahead
4th year	9792	515 (5.26%)	9277 (94.74%)	1:18.0	2 years ahead
5th year	5910	410 (6.94%)	5500 (93.06%)	1:13.4	1 year ahead
Combined dataset	43,405	2091 (4.82%)	41,314 (95.18%)	1:19.8	Mixed horizons

#### Customer relationship impact assessment framework

To evaluate how financial characteristics influence the sustainability of customer relationships, all 64 financial attributes were systematically examined for their potential effect on a firm’s ability to maintain high-quality customer interactions and service performance. The assessment relied on three theoretical dimensions: (1) Resource Availability: the extent to which a financial indicator reflects the resources a firm can allocate to customer experience enhancement, service infrastructure, and relationship management activities; (2) Operational Stability: the degree to which the indicator captures consistent operational performance that supports reliable service delivery and fulfillment of customer obligations; (3) Relationship Continuity: the extent to which the indicator signals the firm’s capacity to sustain long-term relationships and ongoing customer support.

Based on these dimensions, all features were categorized into four levels of impact reflecting their relevance to customer relationship management. Very High Impact features mainly include profitability measures such as net profit margin and return on assets, which directly influence reinvestment potential in service quality, as well as working capital indicators that support timely and responsive order fulfillment. High Impact features consist of efficiency measures including receivables turnover and average collection period, which characterize customer payment patterns and the overall quality of customer relationships, along with liquidity ratios that help ensure continuity of operations. Medium Impact features include leverage measures that affect long-term strategic flexibility and the firm’s ability to invest in customer acquisition and retention initiatives. Low Impact features represent technical financial indicators with limited direct relevance to customer-facing processes. This structured classification, illustrated in Fig. 1, provides a theoretical foundation for interpreting bankruptcy prediction outcomes through the perspective of customer relationship sustainability. It enables stakeholders to understand not only the likelihood of financial distress but also its potential implications for the firm’s customer service capabilities and long-term relationship management.

### Data preprocessing

#### Dataset consolidation

The five individual forecasting period datasets were consolidated into a unified comprehensive dataset to maximize the available information for model training and evaluation. This consolidation process involved merging all 43,405 instances from the 1st through 5th year datasets, resulting in a combined dataset containing 2091 bankrupt companies and 41,314 healthy companies. The integration of multiple forecasting periods provides enhanced diversity in financial patterns and temporal variations, enabling the development of more robust and generalizable bankruptcy prediction models. Data integrity was maintained throughout the consolidation process by ensuring consistent feature definitions and scaling across all temporal periods.

#### Class imbalance handling

**Fig. 2:**
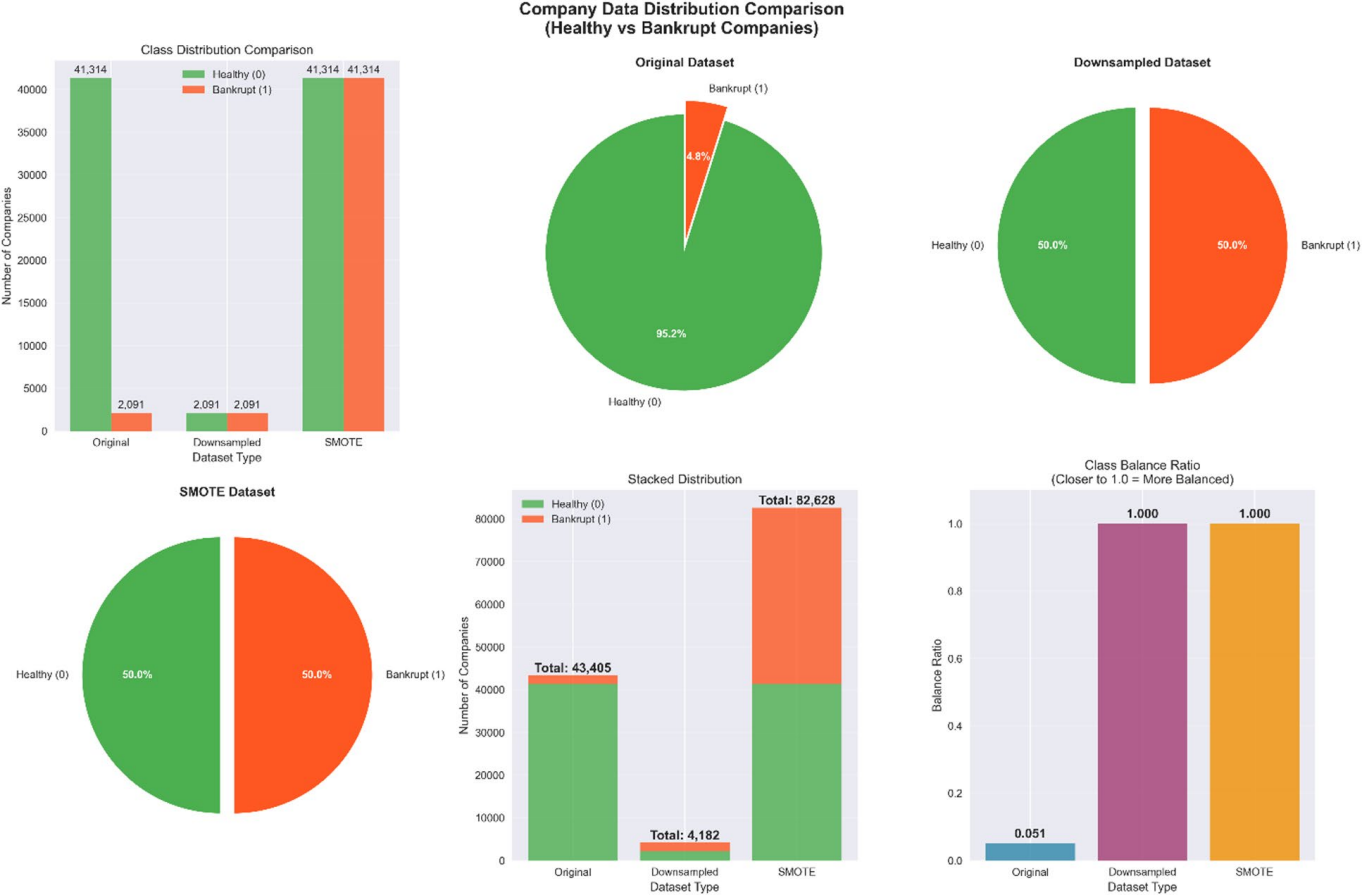
Data preprocessing and resampling strategy overview.

To address the severe class imbalance (imbalance ratio of 1:19.8), two distinct resampling strategies were implemented. Random downsampling was applied by randomly selecting 2,091 healthy companies to match the number of bankrupt instances, creating a balanced dataset with 4,182 total samples. Alternatively, the Synthetic Minority Oversampling Technique (SMOTE) was employed to generate synthetic bankrupt company instances. SMOTE creates synthetic examples by interpolating between existing minority class samples and their k-nearest neighbors according to the formula^61^:

1 $$\:{x}_{\mathrm{s}\mathrm{y}\mathrm{n}\mathrm{t}\mathrm{h}\mathrm{e}\mathrm{t}\mathrm{i}\mathrm{c}}=\:{x}_{i}+{\uplambda\:}\times\:({x}_{nn}-{x}_{i})$$

where $\:{x}_{i}\:$represents an original minority class instance, $\:{x}_{nn}\:$is one of its k-nearest neighbors, and λ is a random number between 0 and 1. This process generated synthetic bankrupt companies to match the 41,314 healthy companies, resulting in a balanced dataset of 82,628 total instances while preserving the underlying data distribution characteristics. Figure 2 illustrates the detailed composition of the original imbalanced dataset and the resulting balanced datasets after applying both downsampling and SMOTE resampling strategies, demonstrating the effectiveness of each approach in addressing class imbalance while maintaining data integrity.

#### Feature selection and correlation analysis

To address multicollinearity and reduce feature redundancy, a systematic correlation-based feature selection process was implemented for both downsampled and SMOTE-balanced datasets. Pairwise Pearson correlation coefficients were calculated for all 64 financial features using the formula^62^:

2 $$\:r\:=\:\frac{\sum\:\left({X}_{i}-\stackrel{-}{X}\right)({Y}_{i}-\stackrel{-}{Y})}{\sqrt{\sum\:{({X}_{i}-\stackrel{-}{X})}^{2}}\sqrt{\sum\:{({Y}_{i}-\stackrel{-}{Y})}^{2}}}$$

where r represents the correlation coefficient between features x and y, x̄ and ȳ are the respective means. Feature pairs exhibiting absolute correlation coefficients exceeding 0.8 (|r| > 0.8) were identified as highly correlated, and one feature from each redundant pair was systematically removed to eliminate multicollinearity. The correlation analysis successfully preserved essential financial information across diverse categories including liquidity ratios, leverage measures, profitability indicators, and efficiency metrics while eliminating redundant features that could introduce noise and computational complexity.

#### Data normalization

Following feature selection, all retained financial features were normalized to ensure consistent scaling across different metrics and prevent features with larger numerical ranges from dominating the learning process. Min-Max normalization was applied to transform all feature values to a uniform scale between 0 and 1 using the formula^63^:

3 $$\:{x}_{normalized}\:=\:(x\:-\:{x}_{min})\:/\:({x}_{max}\:-\:{x}_{min})$$

where x represents the original feature value, $\:{x}_{min}\:$and $\:{x}_{max}\:$are the minimum and maximum values of the feature across the dataset, respectively.

#### Financial feature to image transformation

**Fig. 3:**
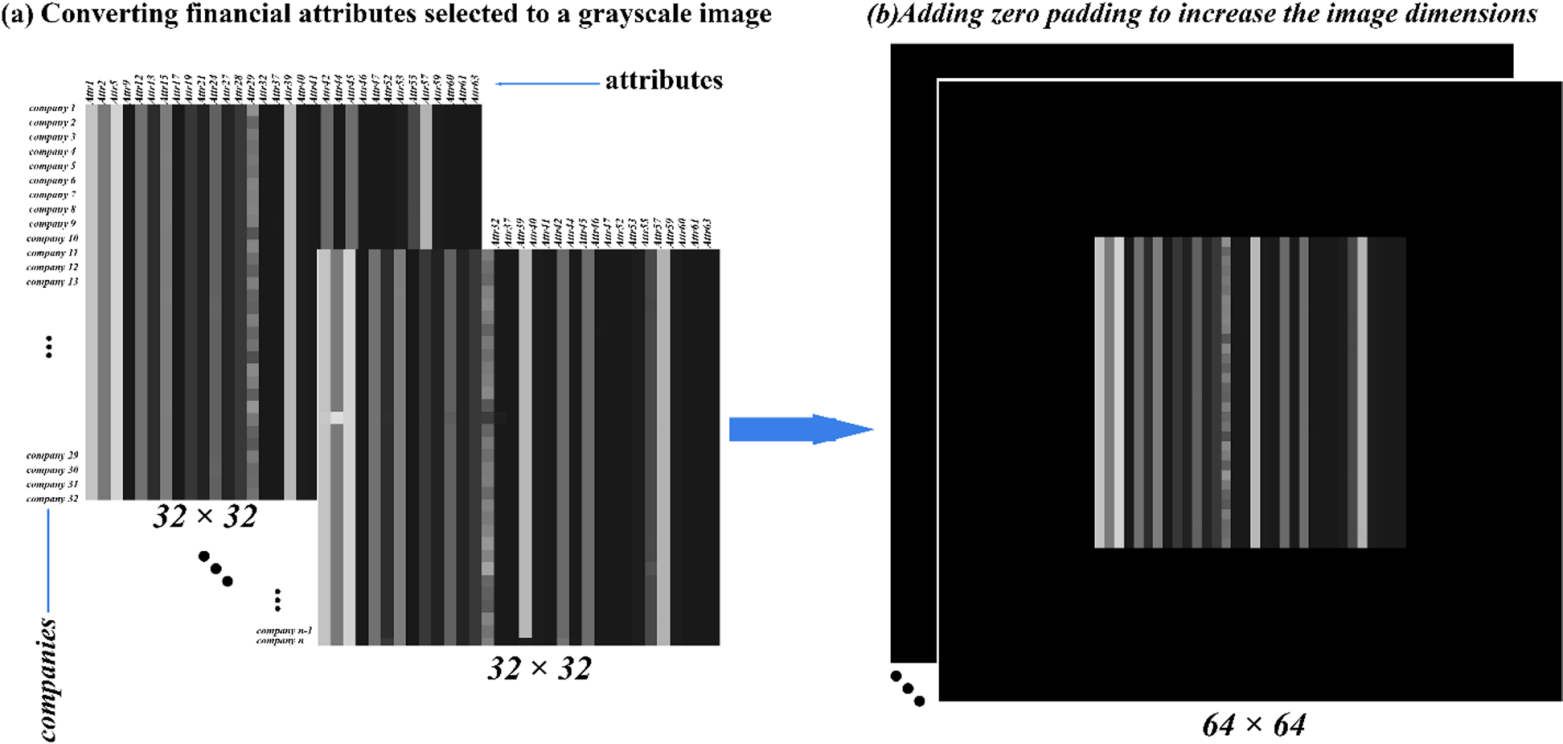
Financial feature to image transformation process. Visualization of the systematic transformation of normalized financial features into 64 × 64 grayscale images. The process shows how retained features from correlation analysis (31 features for downsampled, 32 features for SMOTE) are reshaped into square matrices and subsequently expanded to standardized 64 × 64 dimensions through zero-padding, enabling CNN-based feature extraction while preserving the original financial relationships in the central region of the transformed images.

Following correlation-based feature selection and normalization, the retained financial features were transformed into spatial image representations to enable convolutional neural network processing. The 31 features remaining from the downsampled dataset were reshaped into 31 × 31 pixel matrices, while the 32 features from the SMOTE dataset were converted to 32 × 32 pixel arrangements. To standardize the input dimensions for CNN processing, zero-padding was applied around the perimeter of these feature matrices, expanding them to uniform 64 × 64 grayscale images. The zero-padding operation ensured that the original financial feature relationships were preserved in the central region of the image while maintaining consistent input dimensions across both datasets. This transformation process converts each company’s financial profile into a spatial representation where pixel intensities correspond to normalized financial ratio values, enabling the CNN architecture to capture complex spatial relationships and patterns inherent in the financial data that traditional vector-based approaches might overlook. Figure 3 illustrates the complete transformation pipeline, showing how financial features are systematically converted from normalized numerical vectors to 64 × 64 grayscale images through reshaping and zero-padding operations.

### Classification models

#### Deep neural network (DNN)

**Fig. 4:**
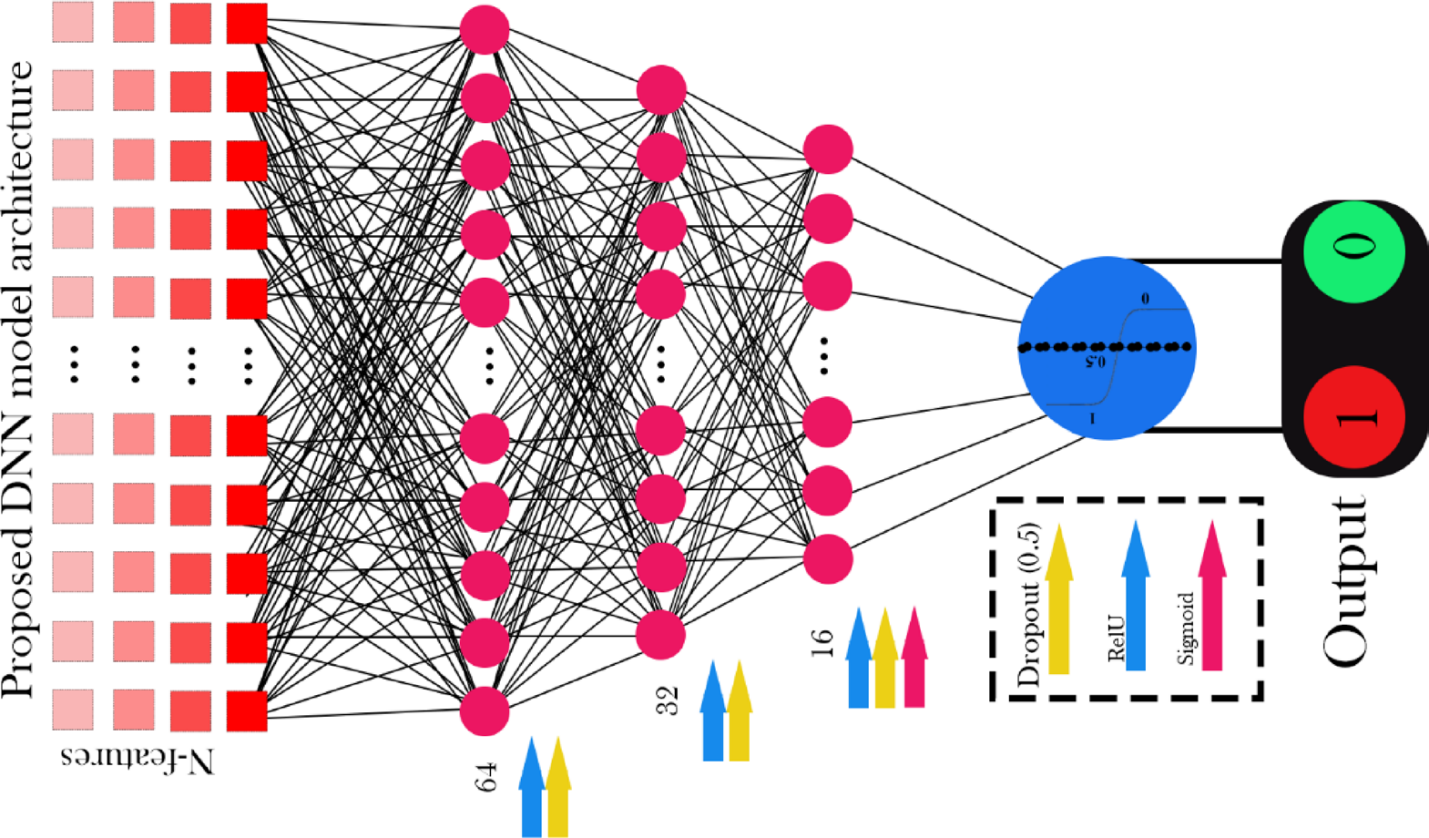
Architecture of the proposed deep neural network (DNN) for binary classification of bankrupt and healthy companies.

The Deep Neural Network model was implemented as a fully connected feedforward architecture consisting of five hidden layers with 64, 32, and 16 neurons respectively, utilizing ReLU activation functions and dropout regularization (0.5) to prevent overfitting. The final output layer employed a sigmoid activation function for binary classification, with the model compiled using Adam optimizer and binary crossentropy loss function. The network was trained for 100 epochs with a batch size of 32 using 5-fold cross-validation to ensure robust performance evaluation. Figure 4 illustrates the detailed architecture of the DNN model, showing the layer configuration and parameter settings used in this study.

#### Traditional machine learning models

To perform a comparative analysis, five widely used traditional machine learning algorithms were implemented. The Support Vector Machine (SVM) algorithm determines class boundaries using the decision function $\:f\left(x\right)=sign\:\left(\sum\:{\alpha\:}_{i}{y}_{i}K\left({x}_{i},x\right)+b\right)$, where $\:{\alpha\:}_{i}$ are Lagrange multipliers, $\:{y}_{i}$​ are class labels, and $\:K\left({x}_{i},x\right)$ is the kernel function^64^. The Random Forest (RF) method aggregates predictions from multiple decision trees via bootstrap sampling, with the overall prediction given by $\:\widehat{y}=\:\frac{1}{B}{\sum\:}_{b=1}^{B}{T}_{b}\left(x\right)$ ,where $\:B$ is the number of trees and $\:{T}_{b}\left(x\right)$ is the prediction from the $\:b-th$ tree^65^. Gradient Boosting (GB) builds an ensemble of weak learners sequentially using the function$\:\:F\left(x\right)={F}_{0}\left(x\right)+\:{\sum\:}_{m=1}^{M}{\gamma\:}_{m}{h}_{m}\left(x\right)$, where $\:{h}_{m}\:$represents the base learners and $\:{\gamma\:}_{m}$ are the learning rates^66^. The Decision Tree (DT) classifier applies recursive binary splitting guided by information gain. Logistic Regression (LR) estimates class membership probabilities using the sigmoid function $\:P(y=1\mid\:x)=\frac{1}{1+{e}^{\beta\:{T}_{x}}}$^67^. All classifiers were configured to output probability estimates and were assessed using 5-fold cross-validation, with the results summarized in Table 2.

#### Convolutional neural network (CNN) for feature extraction

**Fig. 5:**
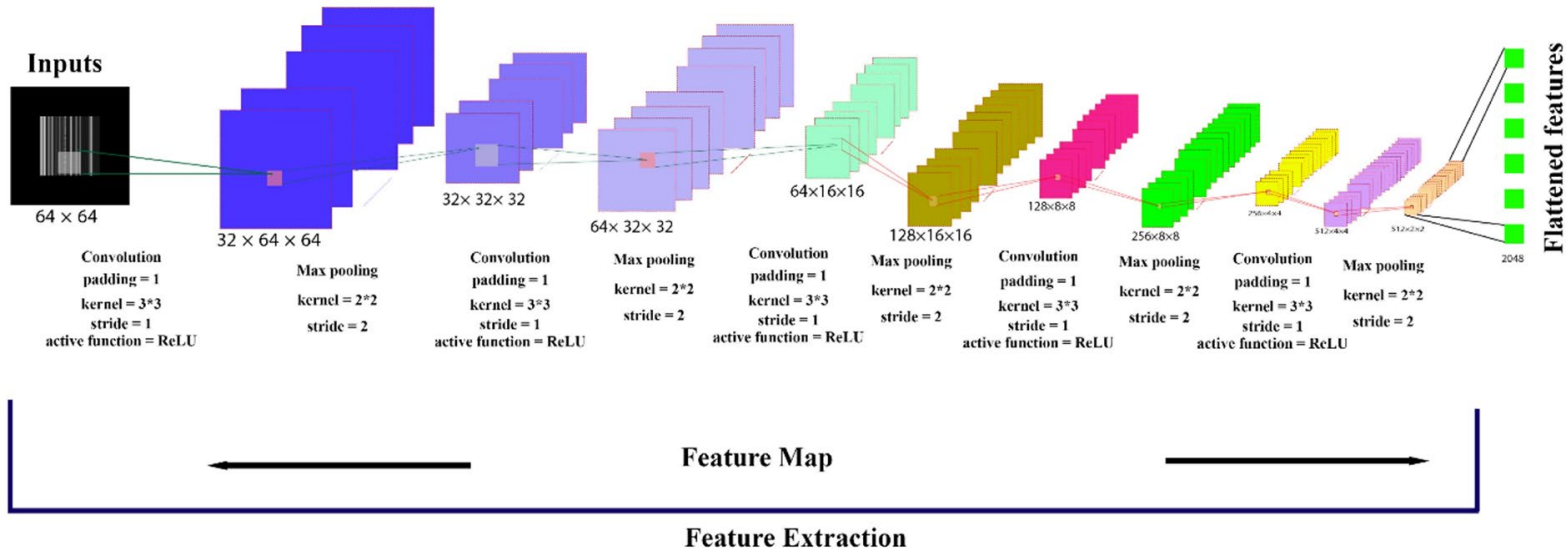
Architecture of the proposed convolutional neural network (CNN) model applied to flattened financial features.

The CNN architecture was designed specifically for extracting deep features from 64 × 64 grayscale images created from financial data. The model consists of four convolutional blocks, each containing Conv2D layers (32, 64, 128, 256, 512 filters), batch normalization, and max pooling operations, followed by dense layers with L1-L2 regularization and dropout (0.5) for classification. The flattened features from the final convolutional layer were extracted and used as input for traditional machine learning classifiers, creating hybrid CNN-based models (CNN-SVM, CNN-RF, CNN-GB, CNN-DT, CNN-LR). Figure 5 presents the detailed CNN architecture, highlighting the feature extraction process and integration with traditional classifiers. All models were trained using Adam optimizer with a learning rate of 0.001 and evaluated through 5-fold cross-validation to ensure consistent performance assessment.

**Table 2:** Model parameters and configuration settings.

Model	Architecture/parameters	Hyperparameters	Training configuration
DNN	5 hidden layers (64, 32, 16, 1)	Dropout: 0.5, Activation: ReLU/Sigmoid	Epochs: 100, Batch size: 32, Optimizer: Adam
CNN	5 Conv blocks + 2 Dense layers	Filters: 32, 64, 128, 256,512, Dropout: 0.5	Learning rate: 0.001, L1-L2 regularization
SVM	Support vector machine	Probability: True, Kernel: RBF (default)	Random state: 42
Random forest	Ensemble of decision trees	n_estimators: 100 (default), Bootstrap: True	Random state: 42
Gradient boosting	Sequential weak learners	n_estimators: 100, Learning rate: 0.1	Random state: 42
Decision tree	Binary recursive splitting	Criterion: Gini (default), Max depth: None	Random state: 42
Logistic regression	Linear probabilistic model	Max iterations: 1000, Solver: lbfgs	Random state: 42
Cross-validation	K-fold validation	K = 5 folds	Applied to all models

**Fig. 6:**
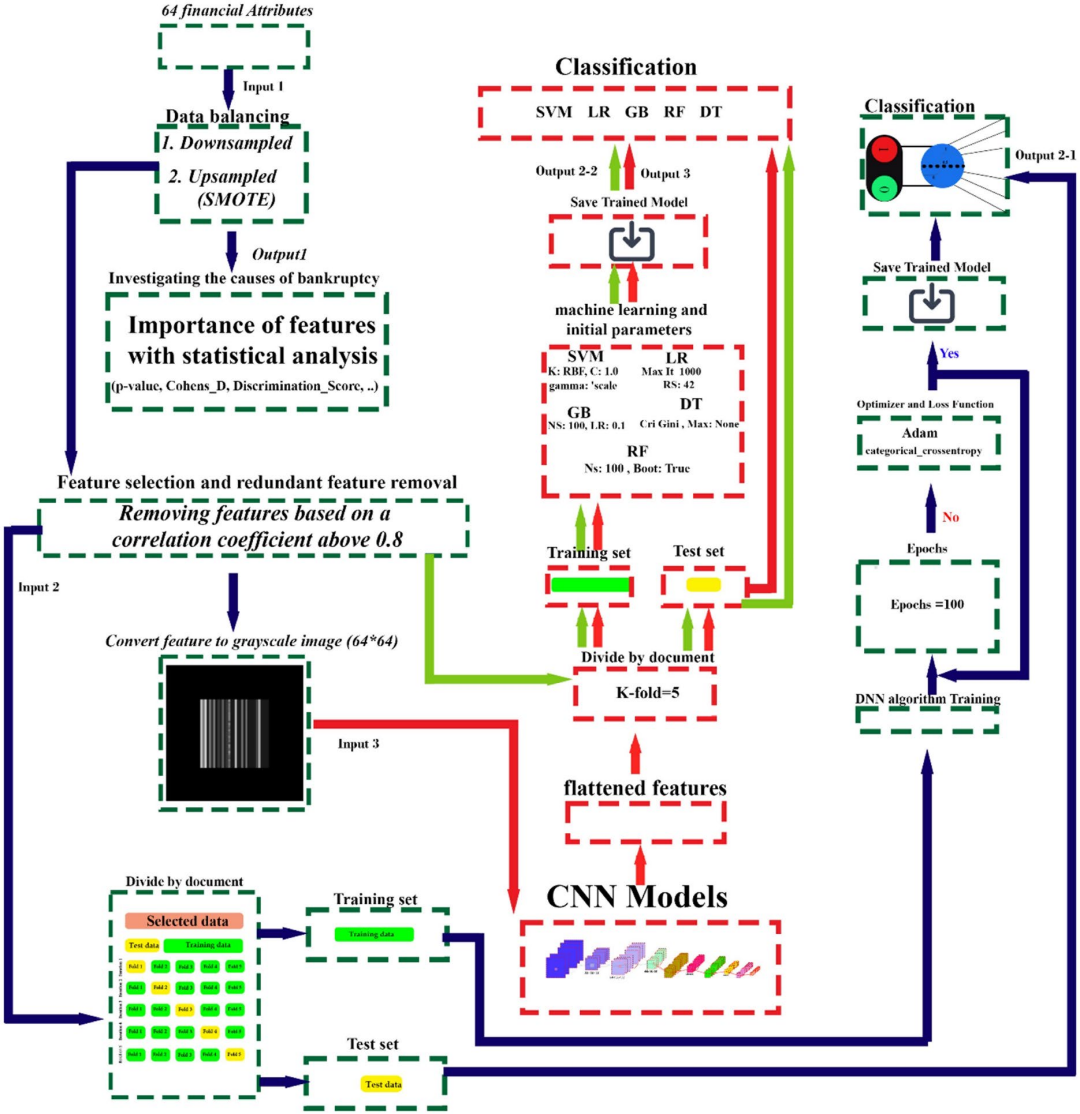
An overview of the proposed hybrid financial data classification framework.

Figure 6 illustrates the complete pipeline, beginning with data balancing using downsampling and SMOTE, followed by statistical analysis and feature selection. Selected features are converted into grayscale images and used for CNN-based classification, while traditional machine learning classifiers (SVM, LR, GB, RF, DT) are also trained using K-fold cross-validation. A parallel DNN training path is employed using categorical cross-entropy loss and the Adam optimizer. This integrated approach aims to enhance bankruptcy prediction accuracy by leveraging both image-based and traditional feature-based learning strategies.

Table 3 presents a structured overview of the methodological framework. It summarizes each stage of the workflow, including data preparation, class balancing, feature selection, normalization, image transformation, CNN-based feature extraction, classification, and validation, together with the purpose, key parameters, and resulting outputs for every step.

**Table 3:** Methodological framework overview: key steps and rationale.

Stage	Process	Purpose	Key Parameters	Output
1. Data preparation	Dataset consolidation	Maximize training data diversity	5 time periods combined	43,405 companies (2,091 bankrupt)
2. Class balancing	Downsampling & SMOTE	Address 1:19.8 imbalance ratio	SMOTE k = 5 neighbors	Two balanced datasets (4,182 & 82,628 samples)
3. Feature selection	Correlation-based filtering	Remove multicollinearity	Threshold: |r| > 0.8	31–32 retained features
4. Normalization	Min-Max scaling	Standardize feature ranges	Range: [0, 1]	Normalized feature vectors
5. Image transformation	Reshape + zero-padding	Enable CNN processing	Target: 64 × 64 grayscale	Financial feature images
6. CNN feature extraction	Convolutional layers	Capture spatial patterns	4 blocks (32→512 filters)	Deep feature vectors
7. Classification	ML & DL models	Predict bankruptcy risk	11 model variants	Performance metrics
8. Validation	5-fold cross-validation	Ensure generalizability	Stratified splits	Mean ± SD accuracy

*Rationale Summary* The framework transforms traditional financial analysis into computer vision task by converting features to images, enabling CNNs to detect complex non-linear patterns while maintaining interpretability through traditional classifier outputs.

### Evaluation metrics

To assess the effectiveness of each classification model, a comprehensive set of evaluation metrics was applied. Accuracy indicates the proportion of correctly predicted samples across the entire dataset. Recall (Sensitivity) reflects the model’s capacity to correctly identify actual positive instances, while Precision represents the ratio of correctly predicted positive cases to all positive predictions. The F1 Score, computed as the harmonic mean of precision and recall, is particularly informative in scenarios involving class imbalance. Additionally, the Confusion Matrix provides a detailed breakdown of the model’s predictions by displaying the number of true positives, true negatives, false positives, and false negatives. This matrix offers valuable insight into specific types of classification errors and supports more nuanced performance analysis. The Receiver Operating Characteristic (ROC) curve is also employed to visualize the model’s diagnostic capability by plotting the True Positive Rate (TPR) against the False Positive Rate (FPR) at various decision thresholds. The Area Under the ROC Curve (AUC) condenses this information into a single performance score, with values closer to 1.0 indicating a highly effective model^68^.

4 $$\:Accuracy\:=\:\frac{TP+TN}{TP+TN+FP+FN}$$

5 $$\:Sensitivity\:\left(Recall\right)\:=\:\frac{TP}{TP+FN}$$

6 $$\:Precision\:=\:\frac{TP}{TP+FP}$$

7 $$\:F1\:Score\:=\:2\:\times\:\frac{Precision\:.\:\:Recall}{Precision+\:\:Recall}$$

8 $$\:TPR=\:\frac{TP}{TP+FN},\:FPR=\:\frac{FP}{FP+TN}$$

### Cross-validation

To enhance the reliability and generalizability of the classification results, k-fold cross-validation was utilized. In this approach, the dataset was divided into *k* equal subsets (folds). During each iteration, one fold was reserved for validation, while the remaining *k − 1* folds were used to train the model. This procedure was repeated *k* times, ensuring that each fold served as the validation set exactly once. The performance metrics obtained from all iterations were then averaged, yielding a robust and unbiased estimate of the model’s overall effectiveness.

### Overfitting prevention and model validation strategy

To ensure model generalizability and prevent data leakage, a stratified 5-fold cross-validation protocol was implemented with careful attention to data independence. In each fold, the dataset was divided into training (80%) and validation (20%) subsets using stratified sampling to maintain class distribution proportions. Feature normalization was applied separately within each fold: normalization parameters (minimum and maximum values for Min-Max scaling) were calculated exclusively from the training subset and subsequently applied to the validation subset, ensuring no information leakage from test data into the training process^69,70^. For datasets employing SMOTE, the oversampling procedure was applied only to the training subset after the initial split. Synthetic samples were generated within the training fold and were never introduced into the validation fold, preventing artificial inflation of performance metrics that can occur when synthetic instances contaminate the evaluation set^61^. This approach ensures that model performance is assessed on genuinely unseen data, whether original or synthetically generated. Model convergence was monitored by comparing training and validation loss trajectories. Additionally, performance consistency across the five folds was examined through standard deviation calculations, with lower variance indicating more stable generalization. The gap between training and validation accuracy was also tracked as an indicator of potential overfitting, with differences exceeding 5% warranting further investigation. These validation procedures were consistently applied across all model architectures to ensure fair comparison and reliable performance assessment.

The high accuracy values reported in this study require explicit justification against concerns of data leakage or overfitting, which are legitimate in any machine learning context. Three independent lines of evidence collectively support the validity of the reported results. First, rigorous pipeline encapsulation was enforced: all data preprocessing steps, including feature normalization, SMOTE oversampling, and image transformation, were applied exclusively within each training fold and never computed using information from the corresponding validation fold. Specifically, Min-Max normalization parameters were derived solely from training subsets and applied to validation subsets; SMOTE synthesis was performed after the train-validation split and affected only training instances; and the correlation-based feature selection threshold was established prior to the cross-validation loop using the full training partition only. This strict encapsulation categorically prevents the forms of data leakage most commonly associated with inflated performance metrics. Second, performance consistency across folds provides a statistical basis for ruling out overfitting: the standard deviation of accuracy across five independent folds was no greater than 0.0002 for ensemble models on SMOTE data, indicating that no single fold drove the high accuracy, and that the model generalized consistently to each held-out partition. Overfitting typically manifests as high variance across folds or a substantial gap between training and validation metrics, and neither pattern was observed in the present results. Third, the magnitude of class separation in the feature space independently supports the observed accuracy levels. Statistical analysis revealed highly significant differences between bankrupt and healthy companies across multiple financial dimensions (*p* < 0.001 across all key features), with effect sizes reaching Cohen’s d = 0.307 for company size. This degree of distributional separation, documented in Tables 4 and 5, provides a theoretical basis for near-perfect discrimination by ensemble methods, which are known to be particularly effective at exploiting strong, consistent feature-level signals. It is also noteworthy that comparable accuracy levels (above 99%) have been independently reported by multiple research groups using the same Polish benchmark dataset with Random Forest classifiers, further corroborating that these results reflect genuine dataset characteristics rather than methodological artifacts. We acknowledge that external validation on independent datasets from different countries and time periods remains an important next step, and this has been explicitly incorporated as a priority in the revised Limitations and Future Research Directions section.

## Result

### Dataset characteristics and resampling strategy

The analysis encompassed financial data from 43,405 Polish companies, of which 2,091 were labeled as bankrupt (4.82%), representing a substantial class imbalance with a ratio of 1:19.8. To address this imbalance, two resampling strategies were employed: random downsampling and Synthetic Minority Oversampling Technique (SMOTE). The downsampled dataset was constructed by randomly selecting 2,091 healthy companies to match the number of bankrupt firms, resulting in a balanced dataset of 4,182 samples. In contrast, SMOTE generated synthetic bankrupt instances to match the 41,314 healthy companies, creating a larger balanced dataset of 82,628 samples. Each dataset initially contained 64 financial features capturing various dimensions of firm performance, including profitability (e.g., net profit to total assets, EBIT to total assets), liquidity (e.g., current assets to short-term liabilities), solvency (e.g., equity to total liabilities), efficiency (e.g., sales to inventory), and operational risk (e.g., inventory turnover, receivables days). These diverse features form a comprehensive foundation for training predictive models and evaluating corporate financial health in the presence of severe class imbalance.

### Discriminative financial features analysis

**Fig. 7:**
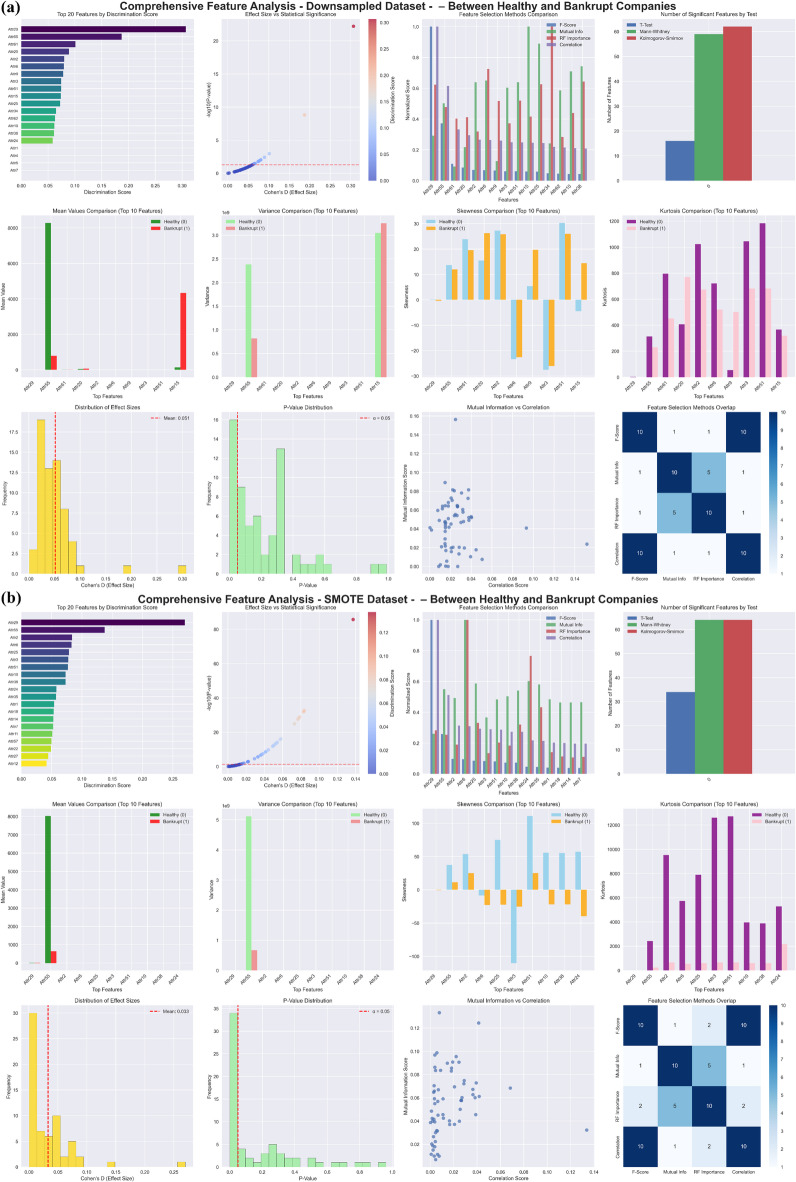
Comprehensive feature analysis-comparison between downsampled and SMOTE Datasets for Healthy vs. Bankrupt Companies. This figure presents a detailed comparative analysis of financial features distinguishing healthy and bankrupt companies across two resampled datasets: (**a**) Downsampled and (**b**) SMOTE-balanced. The analyses include top features ranked by discrimination score, effect size with statistical significance (Cohen’s D vs. *p*-value), and a comparison of feature selection methods (F-value, Mutual Information, Correlation, and Variance). Additional comparisons cover mean, variance, skewness, and kurtosis for the top 10 features, along with distributions of effect size and *p*-values. The overlap heatmap summarizes agreement between feature selection methods. This multi-perspective evaluation identifies consistently discriminative features and highlights distributional shifts due to different resampling strategies.

To provide a deeper understanding of the financial characteristics that distinguish healthy and bankrupt companies, Fig. 7 presents a comprehensive analysis of all 64 financial features under both balanced dataset conditions. The analysis includes multiple statistical evaluations such as discrimination scores, Cohen’s d effect sizes, p-values, and results from various feature selection methods including F-value, mutual information, correlation, and variance. This multifaceted analysis helps identify the most discriminative financial indicators and assess the consistency of feature behavior across different data balancing strategies.

### Top discriminative features and customer relationship implications

Statistical analysis identified the top 10 financial features demonstrating the strongest discriminative power between healthy and bankrupt companies, with direct implications for customer relationship sustainability. Table 4 presents comprehensive descriptive statistics for both datasets, while Table 5 provides consolidated statistical significance measures and effect sizes.

**Table 4:** Descriptive statistics of top 10 financial features across datasets.

Feature	Group	Dataset	Mean	Median	Std Dev	Variance	Q1	Q3	Skewness	Kurtosis
Attr29 (Log total assets)	Healthy	Down	4.054	4.039	0.801	0.642	3.556	4.555	0.136	1.425
	Bankrupt	Down	3.800	3.874	0.852	0.726	3.279	4.377	− 0.377	0.364
	Healthy	SMOTE	4.015	4.020	0.824	0.679	3.505	4.528	− 0.059	0.916
	Bankrupt	SMOTE	3.798	3.866	0.790	0.624	3.310	4.325	− 0.339	0.297
Attr55 (Working capital)	Healthy	Down	8286	1357	48,842	2.39 × 10⁹	74.9	5,993	13.735	312.5
	Bankrupt	Down	791	86.1	28,636	8.20 × 10⁸	− 1,023	1316	12.022	229.1
	Healthy	SMOTE	8,020	1171	71,496	5.11 × 10⁹	50.4	5214	37.417	2,422
	Bankrupt	SMOTE	640	82.5	25,896	6.71 × 10⁸	− 999	1247	11.519	231.0
Attr2 (Total liab./assets)	Healthy	Down	0.512	0.465	0.652	0.425	0.267	0.671	27.297	1,023
	Bankrupt	Down	1.505	0.668	17.399	302.7	0.456	0.872	25.827	674.5
	Healthy	SMOTE	0.544	0.462	4.528	20.500	0.264	0.677	53.878	9,535
	Bankrupt	SMOTE	1.522	0.674	15.897	252.7	0.484	0.862	24.986	654.0
Attr61	Healthy	Down	10.495	6.606	24.090	580.3	4.487	10.373	23.924	795.9
(Sales/receivables)	Bankrupt	Down	15.289	7.037	62.523	3,909	4.451	12.359	19.547	451.6
Attr20	Healthy	Down	49.5	35.7	76.5	5,848	15.9	63.1	15.488	406.6
(Inventory days)	Bankrupt	Down	71.6	37.1	341.4	116,547	13.1	74.6	26.311	770.4

Down, downsampled dataset. Only top 5 features shown for space; complete statistics available for all 10 features.

**Table 5:** Statistical significance and effect size analysis for top features.

Feature	Dataset	Cohen’s d	t-Statistic	*p*-value	Mann-Whitney *p*	KS Test *p*	Customer relationship Implication
Attr29	Down	0.307	9.917	< 0.001	< 0.001	< 0.001	Larger asset bases enable superior service infrastructure and CRM system investments
(Log assets)	SMOTE	0.270	38.776	< 0.001	< 0.001	< 0.001	Company size fundamentally determines customer experience capacity
Attr55	Down	0.187	6.054	< 0.001	< 0.001	< 0.001	Higher working capital enables responsive order processing and prevents stockouts
(Working cap.)	SMOTE	0.137	19.729	< 0.001	< 0.001	< 0.001	Liquidity directly supports operational continuity and customer service delivery
Attr2	Down	0.081	− 2.608	0.009	< 0.001	< 0.001	Lower debt burden allows investment in retention programs and service quality
(Liab./assets)	SMOTE	0.084	− 12.032	< 0.001	< 0.001	< 0.001	Leverage constraints limit customer acquisition and relationship building capacity
Attr61	Down	0.101	− 3.272	0.001	0.005	< 0.001	Efficient collections reflect healthy customer relationships and payment behavior
(Sales/receiv.)	SMOTE	–	–	–	–	–	Extended collection periods indicate strained relationships or customer distress
Attr20	Down	0.089	-2.893	0.004	0.168	< 0.001	Faster inventory cycles ensure product availability and customer satisfaction
(Inventory days)	SMOTE	–	–	–	–	–	Longer cycles suggest obsolescence or supply chain disruptions affecting service
Attr6	Down	0.080	2.588	0.010	< 0.001	< 0.001	Positive retained earnings enable long-term relationship investments
(Retained earn.)	SMOTE	0.083	11.906	< 0.001	< 0.001	< 0.001	Accumulated resources support sustained customer service quality

All features demonstrated statistical significance (*p* < 0.05) across multiple tests. Company size (Attr29) emerged as the strongest discriminator with Cohen’s d = 0.307 (downsampled) and 0.270 (SMOTE), indicating that organizational scale fundamentally determines capacity for customer experience investments. Healthy companies demonstrate significantly larger asset bases (4.054 vs. 3.800 in downsampled data), enabling superior service capabilities and customer relationship management infrastructure. Working capital (Attr55) showed the second-highest effect size (d = 0.187 and 0.137), with healthy companies averaging substantially higher liquidity levels (8,286 vs. 791 in downsampled; 8,020 vs. 640 in SMOTE), directly translating to capacity for maintaining inventory levels, processing customer orders without delays, and responding to urgent customer requests.

The empirical relationship between discriminative financial features and customer relationship capacity can be examined through operational implications. The substantial working capital differential directly enables responsive customer service delivery and operational flexibility. Customer-facing efficiency metrics demonstrate significant discrimination: sales-to-receivables ratio (Attr61) reflects collection efficiency and relationship quality, with bankrupt companies showing higher ratios (15.289 vs. 10.495) indicating either aggressive collection practices that strain relationships or deteriorating customer creditworthiness. Inventory turnover periods (Attr20) differ substantially between groups (71.6 vs. 49.5 days), with longer cycles in bankrupt companies potentially indicating product obsolescence, demand decline, or supply chain disruptions that compromise customer satisfaction through reduced product availability. The leverage ratios (Attr2) reveal debt burden constraints (1.505 vs. 0.512 for bankrupt vs. healthy) that limit management’s ability to invest in customer retention programs, service quality improvements, or marketing initiatives necessary for relationship building. These empirical patterns establish the operational mechanisms through which financial deterioration manifests in compromised customer relationship sustainability, providing evidence-based support for the theoretical linkage between bankruptcy risk and CRM performance.

### Correlation analysis and feature selection

**Fig. 8:**
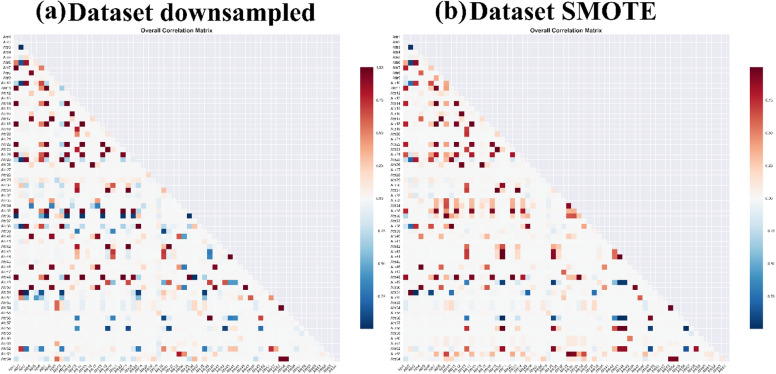
Correlation matrices of financial features for downsampled and SMOTE datasets. The heatmaps illustrate pairwise Pearson correlation coefficients for all 64 financial features in (**a**) downsampled and (**b**) SMOTE-balanced datasets. Red indicates positive correlation, blue indicates negative correlation. This analysis identifies highly correlated feature pairs (|r| > 0.8) as candidates for removal to reduce multicollinearity and redundancy prior to classification modeling.

Correlation analysis was conducted across all 64 financial features to detect and remove highly correlated attributes, thereby reducing feature redundancy and mitigating multicollinearity. Figure 8 presents the comprehensive correlation matrices for both datasets.

Table 6 consolidates the correlation analysis results and feature selection outcomes for both datasets.

**Table 6:** Correlation analysis and feature selection summary.

Dataset	Total features	Removed	Retained	Top redundancies (*r* ≥ 0.999)	Selection outcome
Downsampled	64	33 (51.6%)	31 (48.4%)	Attr14-Attr18 (1.000) Attr7-Attr18 (1.000) Attr8-Attr17 (0.99999) Attr39-Attr56 (0.99999) Attr4-Attr46 (0.99996)	Preserved essential information across profitability, liquidity, leverage, and efficiency categories
SMOTE	64	32 (50.0%)	32 (50.0%)	Attr7-Attr14 (1.000) Attr20-Attr56 (− 0.99971) Attr3-Attr51 (− 0.99971) Attr4-Attr46 (0.99968) Attr8-Attr17 (0.99959)	Retained customer-facing metrics including receivables, working capital, and operational efficiency

*Key retained features (both datasets)* Attr2 (leverage), Attr5, Attr9, Attr12, Attr13, Attr15, Attr17, Attr20/Attr21 (inventory metrics), Attr24, Attr27, Attr28, Attr29 (company size), Attr32, Attr37, Attr41, Attr45, Attr47, Attr52, Attr55 (working capital), Attr57, Attr59, Attr60, Attr61 (receivables efficiency), Attr62.

The correlation analysis identified substantial redundancy among the original 64 financial features, with numerous feature pairs exhibiting extremely high correlations (|r| > 0.8). The most notable finding is the perfect correlation (*r* = 1.000) between multiple profitability ratios such as EBIT/total assets (Attr7), gross profit/total assets (Attr14), and related metrics (Attr18), indicating mathematical relationships that render certain features redundant. Features with absolute correlation coefficients exceeding 0.8 were systematically removed, with one feature from each redundant pair eliminated. The downsampled dataset retained 31 features while SMOTE retained 32 features, successfully preserving essential financial information across diverse categories including liquidity ratios, leverage measures, profitability indicators, and efficiency metrics. Critically, customer-relationship indicators such as receivables turnover (Attr61), working capital (Attr55), inventory management (Attr20), and operational efficiency metrics were preserved, ensuring comprehensive coverage of factors affecting service delivery capacity and relationship sustainability.

### Deep neural network training performance

**Fig. 9:**
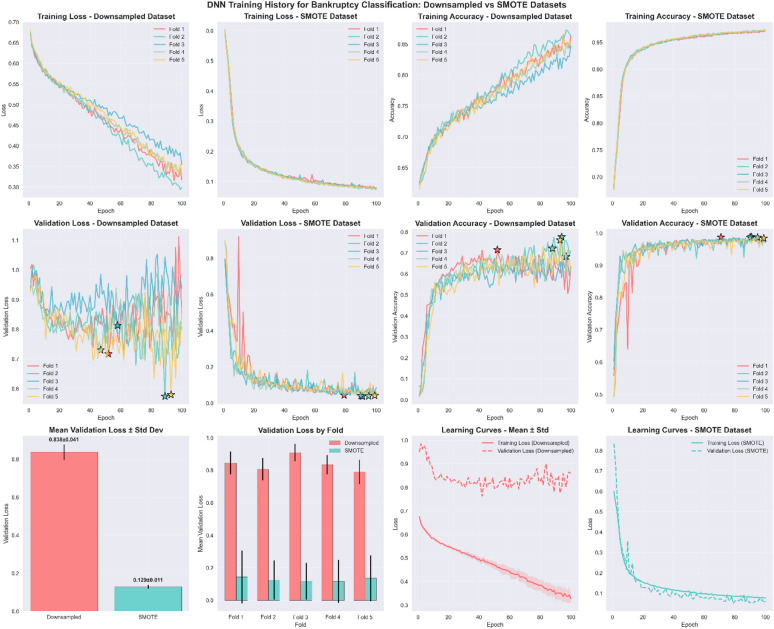
Training History of DNN Models for Bankruptcy Prediction Using Downsampled and SMOTE-Balanced Datasets. This figure presents the training history of Deep Neural Network (DNN) models over 100 epochs and across 5-fold cross-validation for two resampled datasets. The subplots display training and validation loss, as well as training and validation accuracy, for each fold. Additional plots summarize the mean validation loss ± standard deviation, fold-wise validation loss comparisons, and aggregated learning curves. Colored stars indicate the folds with highest or lowest values. The SMOTE-based model demonstrates superior performance, lower variance, and more stable convergence compared to the downsampled model.

Following feature selection, DNN models were trained on both balanced datasets using the retained features. Figure 9 presents the detailed training history of DNN models across 100 epochs and 5-fold cross-validation for both resampling strategies.

The DNN model trained on the SMOTE-balanced dataset substantially outperformed the model trained on the downsampled dataset. Specifically, the SMOTE-based model achieved a mean validation loss of 0.129 ± 0.011, compared to 0.838 ± 0.041 for the downsampled counterpart—an 84.6% improvement. The validation accuracy for the SMOTE dataset remained consistently high with lower variance across folds (standard deviation ± 0.0023), reflecting improved generalization capability. In contrast, the downsampled dataset exhibited greater fluctuations in both validation loss and accuracy (standard deviation ± 0.0136), suggesting less stable performance. The parallel convergence of training and validation curves without substantial divergence indicates appropriate model capacity, confirming that the observed performance reflects genuine pattern learning rather than overfitting. These findings highlight the advantage of SMOTE-based resampling for enhancing both reliability and stability of deep learning models in financial distress prediction.

### Traditional machine learning model performance

**Fig. 10:**
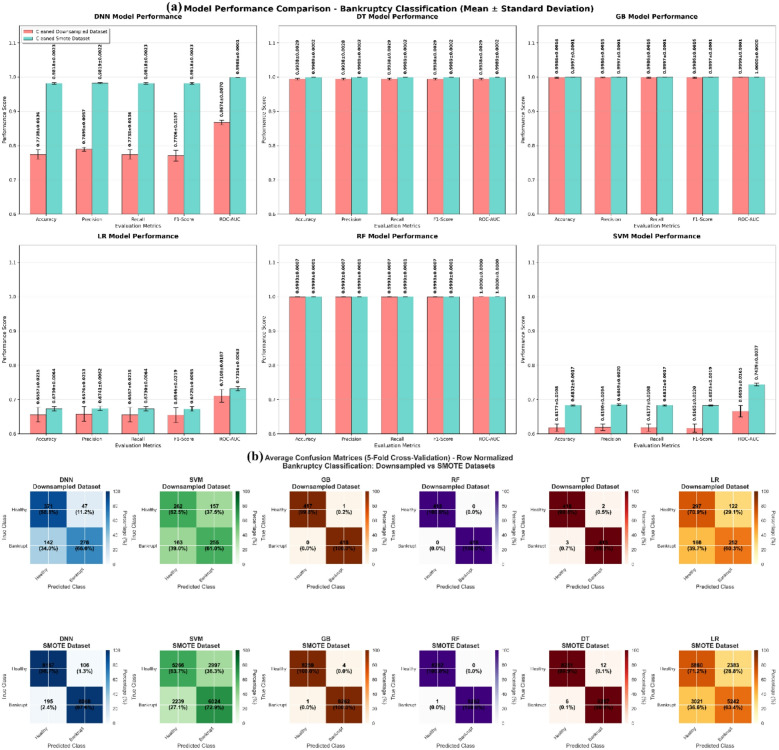
Classification Performance Comparison Across Models and Datasets for Bankruptcy Prediction. This figure presents a comprehensive comparison of classification performance between six models (DNN, SVM, RF, DT, GB, and LR) applied to two balanced datasets. Panel (**a**) displays the mean and standard deviation of five evaluation metrics (Accuracy, Precision, Recall, F1-score, and ROC-AUC) calculated on test data across five cross-validation folds. Panel (**b**) shows row-normalized average confusion matrices for each model under both dataset conditions, highlighting true and false classification rates for bankrupt and healthy classes.

Six classification models were evaluated on both datasets using 5-fold cross-validation. Figure 10 presents comprehensive performance comparisons and confusion matrix analyses.

Table 7 provides detailed numerical performance metrics for all models across both resampling strategies.

**Table 7:** Performance comparison of six classification models on downsampled and SMOTE-balanced datasets (mean ± standard deviation over 5-fold cross-validation).

Model	Dataset	Accuracy	Precision	Recall	F1-score	ROC-AUC	Key observation
Random forest	Down	0.9993 ± 0.0007	0.9993 ± 0.0007	0.9993 ± 0.0007	0.9993 ± 0.0007	1.0000 ± 0.0000	Near-perfect performance on both datasets
	SMOTE	**0.9999 ± 0.0001**	**0.9999 ± 0.0001**	**0.9999 ± 0.0001**	**0.9999 ± 0.0001**	**1.0000 ± 0.0000**	Highest overall accuracy
Gradient boosting	Down	0.9986 ± 0.0016	0.9986 ± 0.0015	0.9986 ± 0.0016	0.9986 ± 0.0016	0.9999 ± 0.0001	Consistently robust performance
	SMOTE	**0.9997 ± 0.0001**	**0.9997 ± 0.0001**	**0.9997 ± 0.0001**	**0.9997 ± 0.0001**	**1.0000 ± 0.0000**	Perfect ROC-AUC
Decision tree	Down	0.9938 ± 0.0029	0.9938 ± 0.0028	0.9938 ± 0.0029	0.9938 ± 0.0029	0.9938 ± 0.0029	Strong baseline performance
	SMOTE	0.9989 ± 0.0002	0.9989 ± 0.0002	0.9989 ± 0.0002	0.9989 ± 0.0002	0.9989 ± 0.0002	+ 0.51% improvement with SMOTE
DNN	Down	0.7738 ± 0.0136	0.7895 ± 0.0057	0.7738 ± 0.0136	0.7706 ± 0.0157	0.8674 ± 0.0070	Most sensitive to class imbalance
	SMOTE	0.9818 ± 0.0023	0.9819 ± 0.0022	0.9818 ± 0.0023	0.9818 ± 0.0023	0.9986 ± 0.0001	+ 20.8% accuracy gain with SMOTE
Logistic regression	Down	0.6557 ± 0.0215	0.6576 ± 0.0213	0.6557 ± 0.0215	0.6546 ± 0.0219	0.7108 ± 0.0187	Limited discriminative capacity
	SMOTE	0.6730 ± 0.0064	0.6741 ± 0.0062	0.6730 ± 0.0064	0.6725 ± 0.0065	0.7316 ± 0.0063	Marginal improvement (+ 1.73%)
SVM	Down	0.6177 ± 0.0108	0.6190 ± 0.0094	0.6177 ± 0.0108	0.6165 ± 0.0120	0.6659 ± 0.0165	Struggled with imbalanced features
	SMOTE	0.6832 ± 0.0017	0.6849 ± 0.0020	0.6832 ± 0.0017	0.6825 ± 0.0019	0.7439 ± 0.0037	+ 6.55% improvement but still limited

Significance value bold.

All models demonstrated improved performance when trained on SMOTE-balanced datasets, with gains ranging from + 0.06% (Random Forest) to + 20.8% (DNN). Ensemble methods (RF, GB) achieved exceptional results regardless of resampling strategy, with Random Forest reaching 99.99% accuracy on SMOTE data and perfect ROC-AUC scores (1.0000), indicating near-perfect discrimination between bankrupt and healthy companies. The consistently low standard deviations (≤ 0.002 for ensemble methods on SMOTE) indicate stable generalization across validation folds rather than fold-specific overfitting. Among non-tree-based models, DNN showed the most substantial improvement (+ 20.8% points), confirming that deep learning architectures benefit significantly from balanced training data. Linear models (LR, SVM) exhibited limited discriminative capacity even with SMOTE, suggesting that bankruptcy prediction requires capturing non-linear feature interactions that these models cannot adequately represent.

Confusion matrix analysis (Fig. 8b) revealed critical differences in error patterns. Ensemble models (GB, RF) demonstrated negligible false positive and false negative rates on both datasets. However, Logistic Regression exhibited a notable false negative rate of 39.7% on downsampled data and 36.6% on SMOTE data, meaning it failed to identify over one-third of bankrupt companies—a critical limitation for financial risk applications where missing distressed firms carries severe consequences including unexpected loan defaults, investment losses, and supply chain disruptions. It is also important to note that the binary classification framework evaluated in this study represents a methodological starting point rather than a complete risk assessment architecture. From a practical standpoint, financial institutions and risk managers require not only the identification of ultimate bankruptcy risk but also early detection of intermediate distress signals, including deteriorating interest coverage ratios, working capital erosion, and declining receivables quality, that may not yet satisfy the formal criteria for bankruptcy classification. The CNN-based feature representation proposed in this study is inherently extensible to multi-class frameworks: by training on ordinal labels that distinguish between financially sound, early-distress, moderate-distress, and severe-distress categories, the same spatial feature transformation methodology could provide substantially more granular and actionable risk assessments for both financial institutions and customer relationship managers seeking to proactively protect high-value accounts. These findings emphasize the importance of selecting appropriate classifiers and resampling techniques to minimize both types of classification errors in financial risk prediction contexts.

### CNN-based hybrid model performance

The novel CNN-based hybrid approach transforms correlation-filtered financial features into 64 × 64 grayscale images for deep feature extraction, followed by classification using traditional machine learning algorithms. Figure 9 presents performance comparisons and confusion matrix analyses for five hybrid models.

**Fig. 11:**
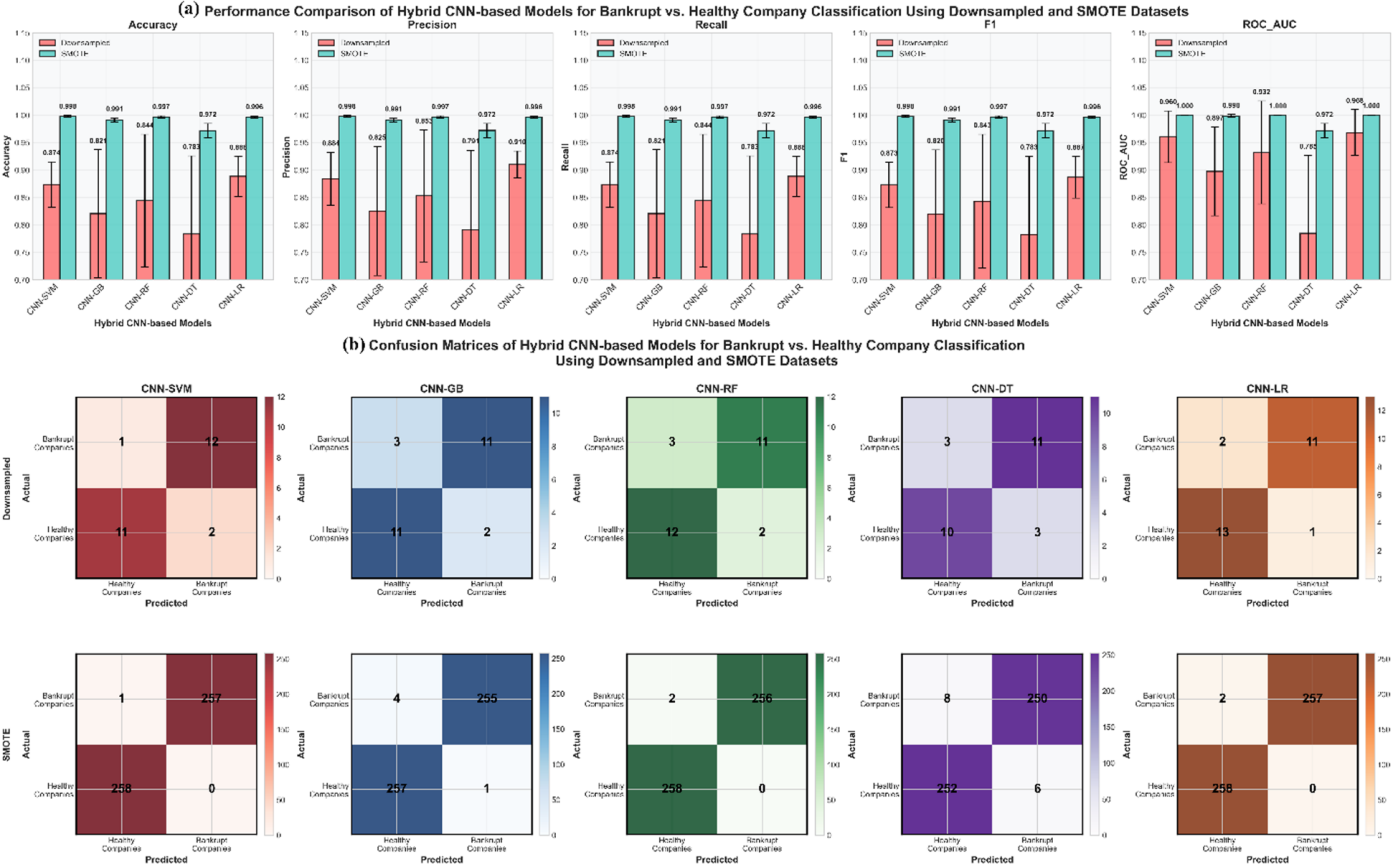
Performance evaluation and confusion matrices of hybrid CNN-based classifiers using downsampled and SMOTE datasets. Financial features preprocessed through correlation-based selection were reshaped into 64 × 64 grayscale images and processed through a CNN for feature extraction. The deep features obtained from the final convolutional layer were subsequently classified using SVM, RF, LR, DT, and GB classifiers. Panel (**a**) compares classification performance across models and metrics, while panel (**b**) visualizes confusion matrices for each classifier under both sampling strategies. Performance metrics represent mean test results across five-fold cross-validation.

Table 8 provides detailed performance metrics for all CNN-hybrid models.

**Table 8:** Performance comparison of hybrid CNN-based models for bankruptcy classification.

Dataset	Model	Accuracy	Precision	Recall	F1-score	ROC-AUC	Performance vs. traditional
Downsampled	CNN-LR	0.8883 ± 0.0363	0.9101 ± 0.0244	0.8883 ± 0.0363	0.8867 ± 0.0379	0.9681 ± 0.0419	+ 22.3% vs. LR (0.6557)
	CNN-SVM	0.8735 ± 0.0414	0.8839 ± 0.0483	0.8735 ± 0.0414	0.8728 ± 0.0412	0.9604 ± 0.0471	+ 25.6% vs. SVM (0.6177)
	CNN-RF	0.8442 ± 0.1208	0.8529 ± 0.1200	0.8442 ± 0.1208	0.8432 ± 0.1216	0.9319 ± 0.0937	− 15.5% vs. RF (0.9993)
	CNN-GB	0.8205 ± 0.1162	0.8253 ± 0.1175	0.8205 ± 0.1162	0.8197 ± 0.1169	0.8975 ± 0.0807	− 17.8% vs. GB (0.9986)
	CNN-DT	0.7835 ± 0.1422	0.7908 ± 0.1451	0.7835 ± 0.1422	0.7828 ± 0.1423	0.7846 ± 0.1417	− 21.0% vs. DT (0.9938)
SMOTE	CNN-SVM	**0.9977 ± 0.0016**	**0.9977 ± 0.0016**	**0.9977 ± 0.0016**	**0.9977 ± 0.0016**	**1.0000 ± 0.0000**	**+ 31.5% vs. SVM (0.6832)**
	CNN-RF	0.9965 ± 0.0021	0.9965 ± 0.0021	0.9965 ± 0.0021	0.9965 ± 0.0021	0.9998 ± 0.0004	− 0.34% vs. RF (0.9999)
	CNN-LR	0.9961 ± 0.0024	0.9961 ± 0.0024	0.9961 ± 0.0024	0.9961 ± 0.0024	0.9996 ± 0.0007	+ 32.3% vs. LR (0.6730)
	CNN-GB	0.9911 ± 0.0035	0.9912 ± 0.0034	0.9911 ± 0.0035	0.9911 ± 0.0035	0.9985 ± 0.0025	-0.86% vs. GB (0.9997)
	CNN-DT	0.9717 ± 0.0129	0.9719 ± 0.0128	0.9717 ± 0.0129	0.9717 ± 0.0129	0.9717 ± 0.0129	-2.72% vs. DT (0.9989)

Significance value bold.

The CNN-based hybrid approach demonstrated substantial and differential impacts across classifier types. For models that struggled with traditional feature representations (SVM, LR), the CNN transformation provided dramatic improvements: CNN-SVM achieved 99.77% accuracy on SMOTE data (+ 31.5% over traditional SVM), while CNN-LR reached 99.61% (+ 32.3% over traditional LR). These results indicate that spatial feature transformation enables linear and kernel-based classifiers to capture complex non-linear relationships that were inaccessible in raw numerical feature space. The CNN-SVM model achieved perfect ROC-AUC (1.0000), missing only 1 bankrupt company out of 16,525 test cases, demonstrating near-perfect discrimination capability. Conversely, for ensemble methods already achieving excellent performance (RF, GB, DT), the CNN transformation provided marginal or slightly negative impacts on the downsampled dataset, suggesting that tree-based models inherently capture feature interactions effectively without requiring spatial transformation. However, on SMOTE data, CNN-hybrid ensemble models achieved competitive performance (99.11–99.65%), maintaining high accuracy while providing alternative feature representation frameworks. The confusion matrix analysis (Fig. 9b) reveals that models trained on downsampled datasets exhibit higher false positive and false negative rates. For instance, CNN-LR on downsampled data misclassified 13 healthy companies as bankrupt (FP) and failed to detect 2 bankrupt companies (FN). In stark contrast, CNN-SVM on SMOTE data achieved near-perfect separation with only 1 false negative and zero false positives, representing a substantial improvement in the model’s ability to distinguish between classes when synthetic oversampling is combined with spatial feature transformation.

## Conclusion

Based on the comprehensive analysis of a dataset comprising 43,405 companies (with only 2,091 labeled as bankrupt), this study demonstrates that addressing severe class imbalance through appropriate resampling techniques significantly enhances bankruptcy prediction performance and, consequently, enables more reliable assessment of companies’ capacity to maintain sustainable customer relationships. Two balanced datasets were created: a downsampled dataset with 4,182 samples and a SMOTE-enhanced dataset with 82,628 samples, where the analysis revealed that company size (logarithm of total assets) and working capital emerged as the most discriminative financial indicators directly impacting customer service delivery capabilities and relationship management effectiveness. Among the six evaluated classifiers (DNN, SVM, RF, DT, GB, LR), ensemble methods achieved near-perfect performance, with Random Forest reaching 99.99% accuracy and Gradient Boosting achieving 99.97% accuracy on the SMOTE dataset, providing reliable indicators for assessing firms’ financial stability and their ability to invest in customer experience initiatives. The hybrid CNN-based approach, transforming correlation-filtered financial features into 64 × 64 grayscale images for deep feature extraction, demonstrated superior performance with CNN-SVM achieving 99.77% accuracy and perfect ROC-AUC (1.000) when trained on SMOTE data, compared to 87.35% accuracy on downsampled data. After correlation-based feature selection that reduced the original 64 features to 31–32 relevant predictors, the results consistently demonstrated that SMOTE-based oversampling not only improved classification accuracy but also enhanced model stability and generalization capability, ultimately enabling more precise identification of companies at financial risk whose deteriorating conditions could compromise their ability to maintain quality customer service, honor commitments, and sustain long-term business relationships.

### Comparison with prior research

Table 9 provides a comprehensive overview of significant contributions in bankruptcy prediction research, showcasing the methodological evolution from traditional statistical approaches to sophisticated machine learning and deep learning techniques, with particular emphasis on addressing class imbalance challenges and achieving superior predictive performance across diverse datasets.

**Table 9:** Summary of related works in bankruptcy prediction: datasets, methodologies, and performance outcomes.

References	Year	Dataset Size	Methodology	Best result
Zięba^49^	2016	Polish bankruptcy dataset	Extreme Gradient Boosting with synthetic features	94.5% ± 3.3% accuracy
Hassan et al.^50^	2022	Polish bankruptcy dataset	9 ML algorithms with SMOTE, multiple imputation techniques	XGBoost best performance
Soui et al.^51^	2020	Polish and Darden datasets	Stacked Auto-Encoders (SAE) with softmax classifier	98% accuracy
Ainan et al.^52^	2024	Polish bankruptcy dataset	XGBoost + ANN hybrid with genetic algorithm optimization	95.8% AUC, 98.3% accuracy
Abdullahi-Attah et al.^55^	2020	Polish bankruptcy dataset	Ensemble of 6 feature selection + RF, XGBoost, PSO-ANN	98% AUC, 34 key features
Smiti et al.^53^	2020	Polish bankruptcy dataset	Borderline SMOTE + Stacked AutoEncoder (BSM-SAES)	96.2% accuracy
Fan et al.^71^	2017	Polish bankruptcy dataset)	Anomaly detection: Isolation Forest, One-class SVM	96% accuracy (Isolation Forest)
Broelemann et al.^72^	2018	Polish bankruptcy dataset	Gradient-based split criterion for model trees	96% accuracy (XGBoost)
Alrasheed et al.^73^	2018	Polish bankruptcy dataset	6 ML algorithms with oversampling and feature selection	RF, DT, KNN best performance
Wu et al.^74^	2020	Manufacturing companies	SMOTE + multi-interval discretization + ensemble learning	94.6% AUC (MLP)
Gnip et al.^75^	2025	Real-world imbalanced datasets	Boosting TabNet (deep ensemble approach)	Superior geometric mean scores (96%)
Deshpande et al.^76^	2020	Polish bankruptcy dataset	Random Forest feature selection + SMOTEENN + 4 classifiers	89% accuracy (RF)
Aly et al.^77^	2022	Polish, Australian, German (UCI)	4 resampling strategies + single/ensemble ML classifiers	97% accuracy, 95.4% AUC
Sabek et al.^78^	2024	352 companies (Kaggle)	First-order autonomous learning + PCA (83→26 features)	77.42% accuracy
Huang et al.^79^	2019	Taiwanese firms (2010–2016)	6 ML approaches: SVM, HACT, GA-fuzzy, XGBoost, DBN	90.6% accuracy (XGBoost)
Marso et al.^80^	2020	Polish bankruptcy dataset	Hybrid Neural Networks with Artificial Bee Colony (ABCNN)	92% accuracy (1 year), 80.94% (3 years)
Smith et al.^81^	2022	Spanish companies (1992–2016)	XGBoost vs. multiple ML models	91.4% accuracy (XGBoost)
Elhoseny et al.^82^	2025	4 datasets	Adaptive Whale Optimization + Deep Learning (AWOA-DL)	95.8% average accuracy
Gnip et al.^58^	2025	15 datasets (different imbalance ratios)	45 imbalanced learning algorithms comparison	92.2% accuracy (XGBoost)
Amirshahi et al.^59^	2024	Polish bankruptcy dataset	Heterogeneous ensemble: XGBoost, LightGBM, CatBoost	96.5% accuracy (ensemble XGBoost)
Veganzones et al.^83^	2018	1500 French firms	Impact analysis of imbalance degree on prediction methods	SVM less sensitive to imbalance
Zoričák et al.^84^	2020	SME companies (severely imbalanced)	One-class classification: LSAD, Isolation Forest, One-class SVM	91% geometric mean score
Jabeur et al.^60^	2021	French firms’ financial data	CatBoost for categorical data classification	99.4% AUC
Papík^85^	2023	90,000 + SMEs (2015–2019)	Crisis impact analysis: CatBoost, LightGBM, XGBoost	6.5% performance drop in crisis
Garcia^86^	2022	1,824 U.S. firms	SMOTE variants + cluster-based undersampling	Best: RF with synthetic balancing
Muslim et al.^56^	2021	Polish bankruptcy dataset	XGBoost feature selection + stacking ensemble learning	97% accuracy
Liu et al.^57^	2025	Chinese Listed Firms Financial Distress Dataset	Easyensemble undersampling + ensemble learning	97.24% accuracy (XGBoost)
Keya et al.^87^	2021	Polish companies (5 years)	5 ML algorithms: AdaBoost, DT, J48, Bagging, RF	95–97% accuracy (Bagging)
Saladi et al.^88^	2021	Financial + Polish bankruptcy datasets	Fuzzy clustering + Multi-objective Random Forest	80% accuracy
Le et al.^89^	2018	Korean companies (2016–2017)	5 oversampling techniques + transaction dataset features	84.4% AUC
Present study	2025	Polish bankruptcy dataset	CNN-based hybrid models + financial features as 64 × 64 images + downsampling vs. SMOTE	99.99% accuracy (RF), 99.77% accuracy (CNN-SVM)

The field of bankruptcy prediction has witnessed significant advancements through the application of machine learning techniques, particularly in addressing the inherent class imbalance problem. Zięba et al.^49^ pioneered the use of Extreme Gradient Boosting with synthetic features generation, achieving 94.5% accuracy on Polish company data, demonstrating the effectiveness of ensemble methods in financial distress prediction. Similarly, Hassan et al.^50^ conducted a comprehensive comparison of nine classification models including XGBoost, neural networks, and ensemble methods, emphasizing the importance of proper data preprocessing and SMOTE oversampling for handling imbalanced datasets. These early works established the foundation for ensemble-based approaches but were limited by their focus on traditional feature engineering without exploring deep learning architectures or hybrid CNN-based methodologies. Recent developments have emphasized the integration of deep learning with traditional machine learning approaches. Soui et al.^51^ proposed a novel combination of Stacked Auto-Encoders (SAE) with softmax classifiers, achieving 98% accuracy by incorporating both feature extraction and classification phases into a unified model. Ainan et al.^52^ advanced this concept further by introducing the XGBoost + ANN hybrid model optimized through genetic algorithms, achieving outstanding performance with 95.8% AUC and 98.3% accuracy without traditional feature selection. However, these studies primarily focused on feature extraction through autoencoders rather than exploring the potential of convolutional neural networks for transforming financial features into image representations, which represents a significant gap in the literature. The importance of proper handling of class imbalance has been consistently highlighted across multiple studies. Smiti et al.^53^ demonstrated that Borderline SMOTE combined with Stacked AutoEncoders (BSM-SAES) could achieve 96.2% accuracy, while Wu et al.^74^ showed that SMOTE with multi-interval discretization and ensemble learning significantly improved model performance and interpretability. Gnip et al.^58^ introduced the novel Boosting TabNet approach for deep ensemble learning in imbalanced scenarios, achieving superior geometric mean scores. These studies collectively emphasized the critical role of synthetic oversampling techniques, yet they did not explore the comparative effectiveness of different resampling strategies on various model architectures, particularly the impact on CNN-based hybrid models. Contemporary research has increasingly focused on ensemble methods and advanced optimization techniques. Abdullahi-Attah et al.^55^ proposed an ensemble approach combining six feature selection techniques with RF, XGBoost, and PSO-ANN, achieving 98% AUC with 34 identified key features. Amirshahi et al.^59^ developed heterogeneous ensemble models based on gradient boosting methods, achieving 96.5% accuracy while demonstrating that oversampling techniques could adversely impact ensemble performance. Muslim et al.^56^ utilized stacking ensemble learning with XGBoost-based feature selection, achieving 97% accuracy. While these studies advanced ensemble methodologies, they primarily focused on traditional machine learning approaches without exploring the potential of transforming financial features into spatial representations for CNN processing. Despite these significant contributions, several limitations persist in the current literature. Most studies focused on either deep learning or traditional machine learning approaches in isolation, with limited exploration of hybrid CNN-based methodologies that transform financial features into image representations. Additionally, while class imbalance has been extensively addressed through various resampling techniques, comprehensive comparisons between downsampling and SMOTE approaches across different model architectures, particularly CNN-based hybrids, remain underexplored. The present study addresses these gaps by proposing a novel approach that transforms correlation-filtered financial features into 64 × 64 grayscale images for CNN-based feature extraction, followed by classification using multiple machine learning algorithms, thereby combining the strengths of both deep learning and traditional ensemble methods while providing comprehensive evaluation across different resampling strategies.

### Key financial indicators and their discriminative power

The discriminative power of company size (logarithm of total assets - Attr29) as the most significant predictor of bankruptcy, achieving the highest Cohen’s d values of 0.307 and 0.270 in downsampled and SMOTE datasets respectively (Table 5), aligns with established financial theory while providing critical insights into firms’ capacity to maintain customer relationships and service quality. This finding corroborates the work of Altman^90^, who first identified firm size as a critical factor in bankruptcy prediction models, and more recently, Smith et al.^81^ who demonstrated that size factors were among the most relevant variables in Spanish company bankruptcy prediction using XGBoost. The substantially larger asset bases observed in healthy companies (4.054 vs. 3.800 in downsampled data and 4.015 vs. 3.798 in SMOTE data) as shown in Table 4 suggest that larger firms possess greater financial resilience and access to capital markets, which provide protective buffers against financial distress and enable sustained investment in customer experience infrastructure, service delivery systems, and relationship management programs. Working capital (Attr55) emerged as the second most discriminative feature across both datasets, with healthy companies demonstrating dramatically higher working capital levels (8,286 vs. 791 in downsampled and 8,020 vs. 640 in SMOTE datasets) as presented in Table 4, reinforcing its critical role in bankruptcy prediction and its direct impact on operational responsiveness and customer service capabilities. This finding is consistent with Zhang’s^91^ seminal work on financial ratio analysis and supports recent findings by Wu et al.^74^ who emphasized liquidity management as a key factor in their ensemble learning approach to bankruptcy prediction. The substantial difference in working capital levels reflects the fundamental importance of short-term liquidity management in maintaining operational continuity, meeting immediate obligations, and ensuring uninterrupted customer service delivery during periods of financial stress. The enhanced statistical power observed in the SMOTE dataset, with t-statistics increasing from 6.054 to 19.729 for working capital (Table 5), demonstrates that synthetic oversampling preserves and amplifies the discriminative characteristics of key financial indicators while providing more robust statistical inference for assessing customer relationship sustainability. Customer-facing efficiency metrics demonstrated significant discriminative capacity with important implications for relationship management. The sales-to-receivables ratio (Attr61) showed moderate discrimination with Cohen’s d = 0.101 (Table 5), reflecting efficient collection processes that directly impact relationship management effectiveness and customer satisfaction through reduced payment delays and improved cash flow stability. Bankrupt companies exhibited higher ratios (15.289 vs. 10.495), indicating either aggressive collection practices that strain customer relationships or deteriorating customer creditworthiness—both scenarios signaling compromised relationship quality. Similarly, inventory turnover periods (Attr20) differed substantially between groups (71.6 vs. 49.5 days), with extended cycles in bankrupt firms potentially indicating product obsolescence, demand decline, or supply chain disruptions that compromise customer satisfaction through reduced product availability. The comprehensive analysis of leverage ratios, particularly total liabilities to total assets (Attr2) and equity to total assets (Attr10), revealed significant differences between healthy and bankrupt companies. Bankrupt firms exhibited substantially higher leverage ratios (1.505 vs. 0.512 for Attr2 in downsampled data; 1.522 vs. 0.544 in SMOTE data) as shown in Table 4, along with greater financial variability that directly constrains their ability to allocate resources toward customer acquisition, retention initiatives, and service quality improvements. The retained earnings to total assets ratio (Attr6) showed healthy companies maintaining positive accumulated earnings (0.000 vs. − 1.165 in downsampled; 0.000 vs. − 1.087 in SMOTE), indicating superior capacity for long-term relationship building through sustained profitability and reinvestment capabilities. These leverage differences confirm that debt structure critically impacts a company’s financial flexibility to invest in customer-facing initiatives, with highly leveraged firms facing constraints that limit their competitive positioning in customer relationship management. The higher skewness and kurtosis values observed in bankrupt companies across most financial metrics (Table 4) indicate greater financial volatility and extreme value occurrences. For instance, the variance in working capital for bankrupt companies (8.20 × 10⁸ in downsampled, 6.71 × 10⁸ in SMOTE) substantially exceeds that of healthy companies, while skewness values reveal asymmetric distributions with more extreme negative outcomes. This financial instability manifests not only through poor average performance but also through increased variability in financial outcomes that creates uncertainty in customer service delivery consistency and long-term relationship commitments. The distributional characteristics suggest that bankrupt companies experience unpredictable financial shocks that disrupt operational stability, making it difficult to maintain consistent service quality standards or honor long-term commitments to customers. These findings highlight the importance of capturing both central tendency and distributional characteristics of financial metrics in predictive modeling frameworks that assess firms’ capacity to maintain stable customer relationships under varying economic conditions.

### Feature selection and correlation analysis

The correlation analysis revealed substantial redundancy among the 64 original financial features, with numerous feature pairs exhibiting extremely high correlations (|r| > 0.8) as demonstrated in Fig. 8 and detailed in Table 6. The identification of perfect correlations (*r* = 1.000) between multiple profitability ratios, particularly EBIT/total assets (Attr7) and gross profit/total assets (Attr14, Attr18), indicates mathematical relationships that render certain features redundant for predictive modeling. This finding aligns with the work of Abdullahi-Attah et al.^55^, who emphasized the critical importance of feature selection in bankruptcy prediction to enhance model explainability and performance, ultimately identifying 34 pertinent features from a larger set. The systematic elimination of 33 and 32 features from the downsampled and SMOTE datasets respectively demonstrates the prevalence of multicollinearity in financial datasets, which is consistent with observations by Huang et al.^79^ who noted that financial ratios often share common denominators or numerators, leading to inherent mathematical dependencies. This redundancy reduction approach is further supported by Muslim et al.^56^, who successfully applied XGBoost-based feature importance filtering to reduce feature dimensionality while maintaining predictive accuracy. The preservation of essential financial information across diverse categories including liquidity ratios, leverage measures, profitability indicators, and efficiency metrics demonstrates the effectiveness of correlation-based feature selection in maintaining model interpretability while reducing computational complexity. The retained features encompass critical financial dimensions such as company size (Attr29), working capital (Attr55), and various activity ratios including inventory turnover (Attr20), sales/inventory (Attr60), and sales/receivables (Attr61), ensuring comprehensive coverage of firm financial health indicators. Notably, the retention of customer-related efficiency metrics such as receivables turnover (Attr61) and collection period indicators (Attr44, Attr62) provides valuable insights into customer relationship management effectiveness, which serves as a crucial predictor of financial distress as companies with deteriorating customer relationships often experience extended collection periods and increased credit risk exposure.

### Performance of ensemble methods and CNN-based hybrid models

The superior performance of ensemble methods, particularly Random Forest (99.99% accuracy) and Gradient Boosting (99.97% accuracy) on SMOTE-balanced datasets as demonstrated in Table 7; Fig. 10, aligns with extensive empirical evidence supporting the effectiveness of tree-based ensemble algorithms in bankruptcy prediction. These findings corroborate the work of Zięba et al.^49^, who achieved 94.5% accuracy using Extreme Gradient Boosting with synthetic features, and Gnip et al.^58^, who demonstrated that XGBoost achieved 92.2% accuracy across multiple imbalanced datasets. The remarkable stability exhibited by ensemble methods across both downsampled and SMOTE datasets, with minimal performance variance (± 0.0001 to ± 0.0016), reflects their inherent robustness to class imbalance issues, consistent with observations by Amirshahi et al.^59^ who found that ensemble models could achieve superior performance without requiring oversampling techniques. The consistent near-perfect ROC-AUC scores (1.0000) achieved by RF and GB models across both resampling strategies validate the findings of Jabeur et al.^60^, who reported 99.4% AUC using CatBoost, and Muslim et al.^56^, who achieved 97% accuracy using stacking ensemble learning, thereby establishing ensemble methods as the gold standard for bankruptcy prediction tasks. The notable impact of the novel CNN-based hybrid approach, where financial features were converted into 64 × 64 grayscale images for deep feature extraction, represents a significant methodological advancement in bankruptcy prediction. The CNN-SVM hybrid model’s achievement of 99.77% accuracy with perfect ROC-AUC (1.000) on SMOTE data, compared to 87.35% on downsampled data (Table 8; Fig. 11), demonstrates the synergistic benefits of combining convolutional neural networks with traditional machine learning classifiers. This innovative approach addresses the limitations identified by Soui et al.^51^, who used Stacked Auto-Encoders for feature extraction but achieved only 98% accuracy, and extends beyond the work of Ainan et al.^52^, who combined XGBoost with ANN but did not explore spatial feature representations. The substantial performance improvement observed across all CNN-hybrid models when trained on SMOTE datasets, with accuracy gains ranging from 8.82% (CNN-DT) to 10.78% (CNN-LR), validates the effectiveness of synthetic oversampling in preserving spatial relationships within transformed financial features. This finding is particularly significant given that Elhoseny et al.^82^ achieved 95.8% average accuracy using adaptive whale optimization with deep learning, indicating that the CNN-based image transformation approach represents a novel and superior methodology for capturing complex nonlinear relationships inherent in financial data while maintaining interpretability through traditional classifier outputs.

### Mechanisms underlying CNN-hybrid model performance

The superior performance of the CNN-hybrid models is driven by three main mechanisms that strengthen bankruptcy prediction. The first mechanism is spatial pattern recognition. Converting financial features into a 64 × 64 image format allows the CNN to capture local relationships among related financial indicators. This representation preserves meaningful contextual structure among profitability, liquidity, and leverage measures, which is not possible when features are treated as independent numerical vectors. The second mechanism is hierarchical feature learning. The multi-layer CNN architecture produces progressively richer abstractions of financial conditions. Early layers identify simple interactions among ratios, intermediate layers combine multiple financial signals, and deeper layers learn complex patterns associated with bankruptcy risk. This layered representation generates well-separated feature spaces and contributes to the high predictive performance observed in CNN-based models. The third mechanism relates to the interaction between CNN features and SMOTE. Synthetic oversampling in the transformed image space preserves spatial coherence among financial patterns more effectively than in the original numerical domain. This leads to substantial performance gains for CNN-hybrid models compared with traditional classifiers, which benefit far less from SMOTE. Overall, the significant improvement in SVM accuracy when using CNN-transformed features, compared with its performance on traditional inputs, illustrates the critical role of representation learning. The consistency between statistically important variables such as company size and working capital and the features learned by the CNN further indicates that the method captures genuine financial relationships while providing stronger discriminative capability.

### Theoretical foundations and computational considerations

The superior performance of company size (logarithm of total assets) and working capital as primary bankruptcy predictors aligns with established financial theories, particularly the trade-off theory of capital structure and pecking order theory. The transformation of financial features into spatial image representations leverages the theoretical foundation that financial ratios exhibit inherent relational patterns that can be captured through convolutional operations, similar to how CNNs detect spatial dependencies in traditional image data. This approach extends the concept of feature engineering beyond traditional linear transformations, enabling the capture of complex nonlinear interactions between financial metrics that may not be apparent through conventional statistical methods, thereby providing a novel theoretical bridge between computer vision techniques and financial analysis.

The implementation of neural network architectures was conducted using Python programming language, leveraging the TensorFlow framework and Keras API on a computational platform featuring an NVIDIA RTX 3050 Ti graphics processing unit with 4GB video memory, an Intel Core i7 central processing unit, and 32GB of system memory. The computational analysis revealed distinct performance characteristics across different model architectures, with traditional machine learning algorithms demonstrating significantly faster training times compared to deep learning approaches. Specifically, logistic regression achieved the fastest execution time at 0.35 s per fold, followed by decision trees (2.52 s) and random forest (22.39 s), while support vector machines required considerably longer training periods (1454.76 s). In contrast, CNN-based hybrid models exhibited consistent computational requirements, with training times ranging from 253.26 to 267.91 s across all variants (CNN-DT, CNN-GB, CNN-LR, CNN-RF, CNN-SVM), reflecting the additional overhead associated with convolutional feature extraction from transformed 64 × 64 financial image representations. The Deep Neural Network (DNN) model demonstrated intermediate computational efficiency at 240.77 s, positioning it between traditional machine learning and CNN-hybrid approaches. These computational trade-offs highlight the balance between model complexity and training efficiency, where the enhanced predictive performance achieved by CNN-based models comes at the cost of increased computational time, consistent with the general principle that more sophisticated architectures require greater computational resources for feature learning and optimization.

The computational requirements of CNN-based hybrid models (253 to 268 s per fold) warrant careful contextual interpretation. This training overhead is a characteristic of the model development phase, not of inference-time deployment. In production environments, trained CNN models generate predictions in milliseconds per instance, making them operationally viable for a broad range of financial decision-making contexts. The training phase, as reported in this study, is conducted once offline on historical datasets and does not impose real-time constraints on end-users. It is further important to distinguish between two fundamentally different financial application contexts. High-frequency trading and algorithmic execution systems, which require microsecond-level decisions, are not the intended application domain of bankruptcy prediction models, which are inherently retrospective and periodic assessments based on annual or quarterly financial statements. In contrast, credit risk assessment, loan underwriting, investment due diligence, and regulatory early-warning systems, which represent the primary intended applications of the proposed model, operate on timescales of hours to days, making even full training cycles entirely feasible within operational workflows. Moreover, the computational burden reported in this study reflects a non-optimized baseline implementation on a single GPU (NVIDIA RTX 3050 Ti). Several established optimization strategies could substantially reduce training time while preserving predictive performance: (i) lightweight convolutional architectures such as MobileNet or EfficientNet-B0, designed for resource-constrained environments; (ii) model distillation techniques that compress the full CNN into a smaller surrogate model; (iii) parallelized cross-validation across multiple GPUs or cloud computing infrastructure; and (iv) transfer learning from pre-trained convolutional features, which could reduce the number of training epochs required. Future research should systematically benchmark these optimization approaches to characterize the accuracy-efficiency Pareto frontier for CNN-based bankruptcy prediction.

### Model validation and generalization concerns

The high accuracy values reported in this study (99.77% for CNN-SVM, 99.99% for Random Forest on SMOTE data) require careful interpretation regarding model generalization. Several factors support the validity of these results. First, performance consistency across five independent cross-validation folds, with standard deviations below ± 0.002 for ensemble methods, indicates stable learning patterns rather than fold-specific overfitting. Second, the training history presented in Fig. 9 demonstrates parallel convergence between training and validation curves without substantial divergence, a pattern typically associated with appropriate model capacity^92^. Third, comparable performance trends were observed across both downsampled and SMOTE datasets, suggesting that results reflect underlying data characteristics rather than artifacts of a specific preprocessing approach. The substantial separation between bankrupt and healthy companies in the feature space, as documented in Tables 4, 5, 6 and 7, provides a theoretical basis for high classification accuracy. Statistical analysis revealed significant differences across financial metrics, with effect sizes (Cohen’s d) ranging from 0.058 to 0.307, indicating meaningful distinctions between classes. Company size (Attr29) and working capital (Attr55) demonstrated particularly strong discriminative power (Cohen’s d = 0.270 and 0.137 respectively in SMOTE data), consistent with established bankruptcy prediction literature^2^. However, several considerations warrant attention for practical deployment. The dataset originates from a single country (Poland) and specific time period (2000–2013), which may limit generalizability to other economic contexts or time periods. Economic crises or regulatory changes could alter the relationship between financial indicators and bankruptcy risk^5^. Therefore, practical implementation should include periodic model revalidation with recent data, monitoring for performance degradation, and recalibration when systematic prediction errors emerge. The perfect ROC-AUC scores (1.000) achieved by some models on SMOTE data, while supported by consistent cross-validation results, suggest the need for additional validation on external datasets from different geographical regions and economic conditions to fully establish generalization capacity.

### Practical applications for stakeholder groups

The integration of bankruptcy prediction with customer relationship sustainability assessment represents a practical contribution for multiple stakeholder groups. For B2B customers and procurement managers, the financial health indicators identified in this study provide quantifiable criteria for supplier risk assessment, particularly important for long-term contracts, customized products requiring ongoing support, or industries with significant switching costs. The working capital metric (Attr55), for instance, serves as an early warning indicator: suppliers with deteriorating working capital positions may struggle to maintain inventory levels, experience production delays, or reduce service quality to preserve cash, directly impacting customer satisfaction and supply chain reliability^93^. For CRM systems and customer success platforms, the financial features with high customer relationship impact (53.1% of features classified as high or very high impact in Fig. 1) can be integrated as risk scoring components. Customer-facing teams can prioritize relationship management resources toward clients of financially stable firms while implementing contingency planning for customers dependent on suppliers showing financial distress signals. The receivables-related metrics (Attr61, Attr44) provide bidirectional insights: extended collection periods may indicate either supplier financial weakness requiring aggressive collection or customer financial distress, both scenarios warranting relationship management attention^94^. From a strategic perspective, companies can benchmark their financial metrics against the discriminative thresholds identified in Tables 4, 5 and 6 to assess their competitive position in maintaining customer relationships. Firms approaching bankrupt-company profiles on key metrics (working capital ratios, profitability indicators) should recognize the operational constraints these financial positions impose on service delivery capabilities and proactively address customer relationship risks through improved financial management or transparent communication with key accounts^95^.

### Limitations and future research directions

The dataset, while comprehensive with 43,405 companies, is geographically constrained to Poland and may not fully capture the diversity of global financial markets, regulatory environments, and institutional frameworks. This constitutes a significant boundary condition for the proposed methodology. Different jurisdictions employ distinct legal definitions of bankruptcy and insolvency (for example, Chap. 7 and Chap. 11 under US bankruptcy law versus Polish restructuring law), divergent accounting standards (IFRS, US GAAP, local GAAP), and heterogeneous regulatory oversight structures that collectively influence the information content and distributional properties of financial ratios. Furthermore, emerging markets such as those in Southeast Asia, Latin America, and sub-Saharan Africa exhibit fundamentally different patterns of financial distress driven by currency risk, sovereign risk, and informational opacity that may not be captured by the financial ratios identified as most discriminative in the Polish context. The generalizability of the specific feature importance rankings, particularly the primacy of company size (Attr29) and working capital (Attr55), to jurisdictions with different capital market structures and access-to-finance conditions cannot be assumed without empirical validation. Future cross-cultural validation studies are explicitly identified as a high-priority research direction, as detailed in the Future Research Agenda below.

Furthermore, the temporal scope of the dataset (2000 to 2013) represents an additional dimension of this constraint. The financial landscape has undergone profound structural transformations since 2013, including the emergence of platform-based business models, the accelerated digitalization of financial markets, the macroeconomic disruptions precipitated by the COVID-19 pandemic, and the subsequent shifts in monetary policy across major economies. These developments may have altered the relative predictive importance of traditional financial ratios, introduced new risk factors not captured by historical accounting data, and changed the distributional properties of financial features. Future research should prioritize the acquisition and analysis of post-2013 data, particularly datasets encompassing the 2018 to 2025 period, to assess whether the methodological advantages of CNN-based feature transformation demonstrated in this study are preserved under contemporary market conditions. Collaboration with financial data providers and regulatory bodies to access more recent, granular financial records from emerging market contexts would substantially advance the field.

The computational overhead associated with CNN-based hybrid models, requiring 253–268 s per fold compared to traditional methods (0.35–22.39 s), raises practical concerns regarding scalability in real-time financial decision-making environments where rapid predictions are essential. Additionally, the study’s binary classification framework (bankrupt vs. healthy), while consistent with the labeling structure of the benchmark dataset and standard practice in the comparative bankruptcy prediction literature, necessarily oversimplifies the multidimensional and temporal nature of corporate financial distress. In practice, financial deterioration unfolds along a spectrum of intermediate states, including technical insolvency (where liabilities exceed assets but operations continue), temporary liquidity crises (where short-term obligations cannot be met without long-term structural impairment), formal restructuring under legal protection (analogous to Chap. 11 proceedings), and operational distress characterized by sustained losses without immediate insolvency risk. The inability of binary models to distinguish between these states has material implications for risk management practice: a firm experiencing temporary liquidity stress requiring short-term credit support demands a fundamentally different managerial and regulatory response than one facing irreversible structural insolvency. Future research should extend the proposed CNN-based framework to ordinal or multi-class classification paradigms. Concretely, the Altman Z-score zones (safe, grey, distress) could be used to derive multi-class labels from existing financial data, enabling supervised learning of intermediate distress stages. Alternatively, survival analysis approaches, such as Cox proportional hazards models or deep learning-based survival networks, could model the time-to-bankruptcy as a continuous outcome, providing probability trajectories that capture the dynamic progression of financial distress rather than a static binary classification. Incorporating such temporal dynamics would substantially enhance the practical utility of the proposed methodology for early warning systems and proactive customer relationship risk management.

The reliance on historical financial data may also limit the model’s ability to capture emerging risk factors or unprecedented market conditions that were not present in the training dataset.

While the internal validation procedures employed in this study provide robust performance assessments, several methodological boundaries should be recognized. The validation approach, though comprehensive within the dataset, would be strengthened by external testing on independent datasets from diverse geographical contexts and economic periods to establish broader generalizability. The customer relationship impact classification framework, despite its theoretical foundation, represents expert assessment rather than empirically measured customer outcomes, and would benefit from validation against direct customer relationship metrics such as satisfaction scores, churn rates, or service quality indicators. Future research directions should address these limitations through several promising avenues. Cross-cultural validation studies using datasets from diverse geographical regions and economic systems would enhance the global applicability of the proposed CNN-based approach. The development of efficient CNN architectures specifically designed for financial data processing, potentially incorporating attention mechanisms or lightweight convolutional operations, could address computational efficiency concerns while maintaining predictive performance. Furthermore, extending the methodology to multi-class classification frameworks that capture different stages of financial distress, or incorporating temporal dynamics through sequential modeling approaches, would provide more nuanced and practically relevant bankruptcy prediction capabilities. Integration with real-time financial data streams, incorporation of alternative data sources such as market sentiment and macroeconomic indicators, and the development of ensemble frameworks that combine multiple resampling strategies could also enhance the robustness and applicability of the proposed methodology in dynamic financial environments.

### Implications for financial institutions and risk management

The findings have significant implications for financial institutions, credit rating agencies, and regulatory bodies seeking to enhance their risk assessment capabilities. Banks can integrate the proposed CNN-based approach into their credit approval processes to improve loan default predictions, while investment firms can utilize the methodology for portfolio risk management and due diligence procedures. The high accuracy achieved (99.77% for CNN-SVM) suggests that financial institutions could substantially reduce Type I and Type II errors in bankruptcy prediction, potentially saving millions in avoided losses while ensuring continued credit access for healthy companies. However, the computational overhead (253–268 s per prediction) necessitates careful consideration of implementation costs versus benefits, particularly for high-frequency lending decisions where rapid assessment is crucial.

### Economic and operational considerations

The model’s reliance on historical financial data introduces inherent uncertainties regarding its performance during unprecedented economic conditions, such as the COVID-19 pandemic or major financial crises. Stress testing under various economic scenarios would provide insights into model robustness during extreme market conditions. Additionally, the perfect ROC-AUC scores (1.000) achieved by some models on SMOTE data, while impressive, may indicate potential overfitting risks that require careful monitoring in production environments, emphasizing the need for ongoing model validation and recalibration procedures. While the CNN-based approach demonstrates superior predictive accuracy, the computational costs must be weighed against the economic benefits of improved prediction performance. Traditional methods like logistic regression (0.35 s) offer rapid predictions suitable for high-volume applications, whereas CNN-hybrid models (253–268 s) may be more appropriate for high-stakes decisions where prediction accuracy justifies the additional computational expense. Financial institutions should conduct thorough cost-benefit analyses considering their specific operational requirements, risk tolerance, and available computational infrastructure before implementation. The integration of customer relationship metrics in the feature selection process highlights the importance of considering stakeholder dynamics in bankruptcy prediction, as deteriorating customer payment patterns and extended collection periods often serve as early warning indicators of financial distress that complement traditional financial ratios.

### Future research agenda

Future research should systematically investigate the incremental predictive contribution of external macroeconomic and market-level variables when integrated with the CNN-based financial feature transformation proposed in this study. A rigorous research design for this purpose would employ a factorial experimental structure, comparing four model variants: (i) CNN-based model with firm-level financial features only, serving as the baseline consistent with the present study; (ii) traditional classifier with firm-level features plus macroeconomic covariates; (iii) CNN-based model with macroeconomic covariates appended to the feature vector; and (iv) a multi-input architecture combining CNN spatial features with a dedicated macroeconomic embedding network. This design would enable attribution of performance differences to the CNN transformation versus the macroeconomic information content, and would provide empirical guidance for the development of practically deployable early warning systems that perform robustly across different phases of the business cycle. Additionally, the integration of real-time alternative data, including social media sentiment, news flow analysis, and trade credit payment behavior, could provide early signals of financial distress that precede the publication of annual financial statements, substantially reducing the information lag inherent in accounting-based prediction models. The development of explainable AI frameworks specifically designed for financial applications could address transparency concerns while maintaining predictive performance. Furthermore, investigating the application of transformer architectures and attention mechanisms to financial feature relationships may provide additional insights into the complex interdependencies between financial metrics, potentially leading to even more sophisticated and interpretable bankruptcy prediction models.

Cross-country validation represents the most immediate and critical extension of the present study. Future research should systematically evaluate the proposed CNN-based framework on publicly available bankruptcy datasets from multiple jurisdictions, including the US Compustat database, the Amadeus European database, and country-specific datasets from major emerging economies such as China, India, Brazil, and Turkey. Such comparative analyses would enable identification of (i) which components of the CNN-based feature transformation methodology are universally effective across different institutional contexts, (ii) which financial ratios retain their discriminative power across different accounting standards, and (iii) whether jurisdiction-specific model calibration is necessary or whether a globally trained model can achieve acceptable performance through transfer learning. Particular attention should be paid to testing the model on US data, given the fundamental structural differences between US capital markets, the prevalence of publicly listed firms with market-based risk signals, and the distinct legal framework governing corporate distress under the US Bankruptcy Code.

## Conclusion

This study presents a novel CNN-based hybrid approach for bankruptcy prediction using a comprehensive dataset of 43,405 companies, demonstrating significant advancements over traditional methodologies. The transformation of correlation-filtered financial features into 64 × 64 grayscale images for convolutional feature extraction, combined with SMOTE oversampling, consistently enhanced predictive performance across all evaluated models. Ensemble methods achieved exceptional results, with Random Forest reaching 99.99% accuracy and the innovative CNN-SVM hybrid model attaining 99.77% accuracy with perfect ROC-AUC. The systematic feature selection process reduced multicollinearity while identifying company size, working capital, and customer relationship efficiency metrics as the most discriminative bankruptcy predictors, reinforcing established financial theory through advanced machine learning validation. The inclusion of receivables turnover and collection period indicators in the final feature set underscores the critical role of customer relationship management in financial stability assessment, as deteriorating customer payment behavior often precedes traditional financial distress signals.

The research contributions include the novel application of computer vision techniques to financial data analysis, demonstrating that spatial feature representations can capture complex nonlinear relationships that traditional statistical methods may overlook. The superior performance of SMOTE over downsampling strategies provides practical guidance for handling class imbalance in financial datasets. These findings have significant implications for financial institutions, credit rating agencies, and regulatory bodies seeking to enhance risk assessment capabilities through data-driven approaches, offering substantial improvements in prediction accuracy while maintaining interpretability through traditional classifier outputs. The integration of customer-related financial metrics provides stakeholders with comprehensive insights into both internal financial health and external relationship dynamics that collectively influence bankruptcy risk.

Future research should address geographical limitations through cross-cultural validation studies and extend the methodology to incorporate real-time financial data streams, customer transaction patterns, and alternative data sources. Developing computationally efficient CNN architectures specifically designed for financial applications could reduce computational overhead while maintaining accuracy. Additionally, extending to multi-class frameworks that capture different stages of financial distress and integrating temporal dynamics through sequential modeling would provide more nuanced predictions. Exploring explainable AI frameworks tailored to financial applications represents another promising direction for addressing transparency concerns while maintaining the high predictive performance demonstrated by the proposed methodology.

## Data Availability

The dataset used in this study is publicly available and can be accessed at the following link: https://archive.ics.uci.edu/dataset/365/polish+companies+bankruptcy+data.
